# Effects of gut-derived endotoxin on anxiety-like and repetitive behaviors in male and female mice

**DOI:** 10.1186/s13293-018-0166-x

**Published:** 2018-01-19

**Authors:** Christopher T. Fields, Benoit Chassaing, Alexandra Castillo-Ruiz, Remus Osan, Andrew T. Gewirtz, Geert J. de Vries

**Affiliations:** 1 Neuroscience Institute, Georgia State University, Atlanta, GA 30303 USA; 2 Institute for Biomedical Sciences, Center for Inflammation, Immunity & Infection, Georgia State University, Atlanta, GA 30303 USA; 3 Department of Mathematics and Statistics, Georgia State University, Atlanta, GA 30303 USA

**Keywords:** Lipopolysaccharide, Toll-like receptor 4, (+)-Naloxone, LPS-RS, Anxiety-like behavior, Repetitive behavior, Open field test

## Abstract

**Background:**

Gut dysbiosis is observed in several neuropsychiatric disorders exhibiting increases in anxiety behavior, and recent work suggests links between gut inflammation and such disorders. One source of this inflammation may be lipopolysaccharide (LPS), a toxic component of gram-negative bacteria. Here, we (1) determine whether oral gavage of LPS, as a model of gut-derived endotoxemia, affects anxiety-like and/or repetitive behaviors; (2) test whether these changes depend on TLR4 signaling; and (3) test the extent to which gut-derived endotoxin and TLR4 antagonism affects males and females differently.

**Methods:**

In experiment 1, male wild-type (WT) and *Tlr4−/−* mice were tested for locomotor, anxiety-like, and repetitive behaviors in an automated open field test apparatus, 2 h after oral gavage of LPS or saline. In experiment 2, male and female WT mice received an oral gavage of LPS and an injection of one or two TLR4 antagonists that target different TLR4 signaling pathways ((+)-naloxone and LPS derived from *R*. *sphaeroides* (LPS-RS)). Univariate and multivariate analyses were used to identify effects of treatment, sex, and genotype and their interaction.

**Results:**

In experiment 1, oral gavage of LPS increased anxiety-like behavior in male WT mice but not in *Tlr4−/−* mice. In experiment 2, oral gavage of LPS increased anxiety-like and decreased repetitive behaviors in WT mice of both sexes. Neither antagonist directly blocked the effects of orally administered LPS. However, treatment with (+)-naloxone, which blocks the TRIF pathway of TLR4, had opposing behavioral effects in males and females (independent of LPS treatment). We also identified sex differences in the expression of interleukin-6, a pro-inflammatory cytokine, in the gut both in basal conditions and in response to LPS.

**Conclusion:**

In spite of the ubiquitous nature of LPS in the gut lumen, this is the first study to demonstrate that intestinally derived LPS can initiate behavioral aspects of the sickness response. While an increased enteric load of LPS increases anxiety-like behavior in both sexes, it likely does so via sex-specific mechanisms. Similarly, TLR4 signaling may promote baseline expression of repetitive behavior differently in males and females. This study lays the groundwork for future interrogations into connections between gut-derived endotoxin and behavioral pathology in males and females.

**Electronic supplementary material:**

The online version of this article (10.1186/s13293-018-0166-x) contains supplementary material, which is available to authorized users.

## Background

Dysbiosis of the gut microbiota, defined as a shift toward pathological, pro-inflammatory microbial species, has been linked to a number of neuropsychiatric disorders associated with increased expression of anxiety behavior, including autism [1–3], ADHD [4], and psychological pathologies comorbid with inflammatory bowel disease (IBD) [5, 6]. Microbiota-induced gut inflammation may mediate these behavioral pathologies. An important agent in these effects is likely to be lipopolysaccharide (LPS), a pathogenic component of gram-negative bacteria, which is endogenous to the gut microbiota [7]. When injected systemically, LPS produces well-documented behavioral alterations collectively called “sickness behavior,” which includes an increase in anxiety-like behaviors [8, 9] and suppression of compulsive and repetitive behaviors [10–12].

Intraperitoneal injections of LPS allow direct exposure of LPS to extra-intestinal peritoneal leukocytes that produce systemic cytokines that will provoke a sickness response. Likewise, intravenous injections of LPS facilitate its fast and robust interaction with splenic immune cells and circulating leukocytes. However, it is unknown whether elevations of serum LPS levels originating from gut barrier dysfunction, observed in rodent models of gut dysbiosis (such as emulsifier-fed mice [13], mice with dextran sodium sulfate (DSS)-induced colitis [14, 15], high-fat diet-fed mice [16], and toll-like receptor 2 knockout (*Tlr2−/−*) mice [17]), are responsible for increases in anxiety-like behavior observed in these models. These studies reliably demonstrate a 2- to 3-fold increase in serum LPS levels in experimental subjects relative to controls, a condition termed “metabolic endotoxemia” [18]. As even a 10-μg/kg dose of LPS (10 times lower than in most published studies) is sufficient to increase serum levels of LPS to 25× above baseline [19], it is questionable whether intraperitoneal injections recapitulate the dynamics of LPS-induced inflammation observed in “metabolic endotoxemia.” Furthermore, the site of action may make a difference, as an inflammatory stimulus injected intraperitoneally may differ in its neurobehavioral effects from an inflammatory stimulus administered orally.

Under most circumstances, LPS present on gut bacteria does not cause pathology. However, increased intestinal loads of LPS may breach the intestinal lining, activate intestinally associated innate immune cells, and produce metabolic endotoxemia [18]. Elevated gut levels of gram-negative bacteria have been reported in clinical populations, such as children with autism or individuals with celiac disease [20, 21]. Furthermore, the severity of gastrointestinal conditions correlates positively with levels of anxiety behavior in autistic children [22–25]. In addition, elevated fecal levels of LPS are reported for rodent models of diseases such as diet- and emulsifier-induced obesity, as well as colitis; microbiota transfer from each of these disease models into control subjects causes similar immune and/or behavioral deficits to those observed in the respective disease model [16, 26–28]. However, whether gut-derived LPS influences anxiety behavior remains untested.

The purpose of this study is to (1) determine whether oral gavage of LPS, as a model of gut dysbiosis, affects anxiety-like and/or repetitive behaviors; (2) test whether these changes depend on TLR4 signaling; and (3) test the extent to which gut-derived endotoxin and TLR4 antagonism affects males and females differently. Here, we show that LPS triggers behavioral changes in males as well as females, but the underlying signaling mechanisms may differ. Furthermore, the effects of gut-derived LPS may not depend on systemic TLR4 signaling.

## Methods

### Animals

Three-month old C57Bl/6J mice were used to test the effects of gut-derived LPS on anxiety-like and repetitive behaviors. For experiment 1, 14 male wild-type (C57Bl/6J) mice and 8 male *Tlr4−/−* (*Tlr4*^*lps-del*^ on C57Bl/6 background) mice were randomly selected from a colony bred in-house. Founder mice for this colony were sourced from Jackson Labs (Bar Harbor, ME). As this colony contained a negligible number of females, female subjects were not used in this experiment. For experiment 2, 64 male and 64 female C57Bl/6J mice were purchased at 10 weeks of age (Jackson Labs) and housed in our facility for 2 weeks prior to the study. For both experiments, all subjects were housed in same-sex pairs prior to the beginning of the study. The mice were housed in a room maintained in a 12:12 light-dark cycle, at 68–72 °F and approximately 50% humidity, and were fed ProLab 5001 diet ad libitum (LabDiet, St. Louis, MO). All experimental protocols were approved by the Institutional Animal Care and Use Committee at Georgia State University and were performed in accordance with the National Institutes of Health Guidelines for the Care and Use of Laboratory Animals. All procedures were designed to minimize subject discomfort and use the fewest animals necessary for statistical analysis.

### Experiment 1

On the day of testing, mice were single-housed and fasted for 2 h to ensure gastric emptying [29] and were then administered 300 μg/kg LPS in a total volume of 200 μl saline (LPS from *Escherichia coli* [O111:B4]; Sigma, St. Louis, MO) or saline alone by oral gavage (7 per treatment group for WT mice and 4 per treatment group for *Tlr4−/−* mice). The tip of the gavage needle was dipped in a 30% sucrose solution to decrease gavage-related stress response [30]. Two hours after gavage treatment, subjects were transferred to an automated open field apparatus for 10 min to measure locomotor parameters (ambulatory episodes, ambulatory counts, ambulatory time, ambulatory distance, resting time, and average velocity), anxiety-like behaviors (time spent in the center zone, zone entries, number of rears, and time spent rearing), and repetitive behaviors (time spent in stereotypic circling, number of stereotypic counts, jump counts, jump time, number of clockwise reversals, and number of counterclockwise reversals). After behavior testing, serum samples were collected by terminal cardiac puncture blood collection under isoflurane anesthesia.

### Experiment 2

To determine which TLR4 signaling cascade is responsible for the anxiogenic effects of orally administered LPS, we used (+)-naloxone (NIDA Drug Supply), which blocks the TLR4/TRIF cascade and has low affinity for mu-opioid receptors (1/1000 to 1/10000 the affinity of (−)-naloxone for mu-opioid receptors) [31, 32], and LPS-RS Ultrapure (InVivoGen, San Diego, CA; LPS derived from *Rhodobacter sphaeroides*, hereafter simply referred to as LPS-RS), which blocks the TLR4/MyD88 cascade [33]. LPS molecules, sourced from different bacterial species and strains, differ in level of immunogenicity, ranging from TLR4 agonists, such as *E. coli*-derived LPS, that produce robust inflammation to TLR4 antagonists, such as LPS derived from *R*. *sphaeroides* (LPS-RS), that block the inflammatory effects of pro-inflammatory LPS species [34, 35]. We selected a dose of 60 mg/kg of (+)-naloxone, administered 30 min prior to LPS challenge, as applying this dose and timing blocks sedation and motor impairments induced by acute exposure to ethanol, a condition associated with increased intestinal permeability [36, 37]. We selected a dose of 800 μg/kg of LPS-RS, injected 30 min prior to oral gavage of LPS, as intrathecal injection of this dose and timing has been demonstrated to block neuropathic pain induced by LPS [38].

To ensure gastric emptying, male and female mice were single-housed and fasted for 2 h [29]. Ultimately, eight mice of each sex were assigned to each 2 (gavage treatment) × 2 ((+)-naloxone treatment) × 2 (LPS-RS treatment) group. Ninety minutes into the fast, mice received 60 mg/kg (+)-naloxone in 200 μl saline, 800 μg/kg LPS-RS in 200 μl saline, (+)-naloxone and LPS-RS together in 200 μl saline, or 200 μl saline by intraperitoneal (i.p.) injection. Thirty minutes later, subjects received saline or 300 μg/kg LPS by oral gavage. As in experiment 1, the tip of the gavage needle was dipped in a 30% sucrose solution prior to insertion. Two hours after the oral gavage, mice were tested on an automated open field apparatus as described for Experiment 1. Directly after behavior testing, mice were euthanized for serum and intestinal tissue collection.

### Serum LPS

Hemolysis-free serum was generated by centrifugation of blood using serum separator tubes (Becton Dickerson, Franklin Lakes, NJ). Serum was stored at − 20 °C in silanized tubes. On the day of analysis, serum was diluted 1/40 in LPS-free saline and residual plasma proteins were degraded via a 10-min 70 °C incubation [17]. Serum LPS concentrations were determined using a kit based on a Limulus amebocyte extract (GenScript, Piscataway, NJ) according to manufacturer’s instructions, with samples run in duplicate.

### RT-qPCR for intestinal tissue

We examined the expression of pro-inflammatory (IL-1β, IL-6, TNF-α) and anti-inflammatory (IL-10) cytokines in the gut in response to oral LPS exposure. To do so, 1 in. of jejunum tissue was homogenized in TRIzol (Invitrogen, Carlsbad, CA) for RNA extraction. Reverse transcription was performed with a SuperScript IV First-Stand Synthesis Kit (Invitrogen) in a thermal cycler (Applied Biosystems Inc., Foster City, CA) and real-time PCR was performed in the LightCycler 96 System (Roche, Mannheim, Germany) using FastStart Essential DNA Green Master Kit (Roche) according to the manufacturer’s instructions and as previously described (Castillo-Ruiz et al. 2017). Primers used targeted messenger RNA for *IL-10*, *IL-1β*, *TNF-α*, *IL-6*, *and glyceraldehyde 3-phosphate dehydrogenase* (*GAPDH*) as reference gene (all validated primers from Qiagen Inc., Valencia, CA).

### Luminex cytokine assay

BioRad (Hercules, CA) 4-plex mouse Luminex kits were used to measure serum cytokine levels. The cytokines assayed were TNF-α, IL-1β, IL-6, and IL-10. Assays were performed according to the manufacturer’s instructions, and samples were run in duplicate.

### Statistical analyses

Using SPSS (version 23), univariate (ANOVA) and multivariate analysis of variance (MANOVA) were performed on the data obtained from the automated open field test apparatus. Following ANOVAs, planned contrasts were performed on individual outcome variables to identify directionality between group differences. Multivariate statistics are useful to detect relationships between outcome variables and identify syndromes of behavioral effects, particularly in outcomes with statistically non-significant univariate ANOVAs [39]. Like ANOVA, which tests whether mean differences between groups on a single dependent variable occur by chance, MANOVA tests whether mean differences for a combination of dependent variables occur by chance. Discriminant analysis ranks outcome variables by their contribution to group separation along the combination of all dependent behavioral variables used in the ANOVA analyses. The same group of behavioral measures was used in all discriminant analyses across experiments 1 and 2. Only behavioral measures that differentiate the discriminant functions are listed in the structure matrix. Discriminant functions were validated with both original group case classification tests and leave-one-out cross-validation tests, which gives a more unbiased estimate of the generalizability of the discriminant functions [40, 41].

Effect sizes for sex, genotype, and treatment effects were reported as sample means with 95% confidence intervals. In addition, using SPSS, partial eta squared (“partial *η*^2^”) were reported as effect size measurements of variance within the ANOVA and MANOVA tests. Estimation of population means and 95% confidence intervals were calculated for WT males across experiments 1 and 2 using random effects meta-analysis. All confidence interval estimates were calculated with Exploratory Software for Confidence Intervals or ESCI [42, 43].

Two-way ANOVAs (sex by treatment) were performed for the analysis of gut cytokine expression using GraphPad Prism version 6.01. Significant effects were followed by Fisher’s LSD tests.

## Results

### Experiment 1: role of TLR4 in behavioral response to oral gavage of LPS

**Fig. 1:**
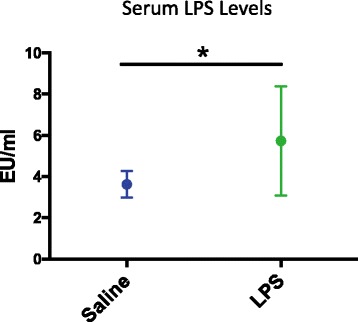
Oral gavage of LPS produces low-grade endotoxemia. Serum LPS levels in WT mice treated with saline (blue bar) or LPS (green bar). LPS treatment significantly increased LPS levels 1.5-fold, 2 h after gavage. Bars indicate mean and 95% confidence intervals. *p < 0.05

In line with other models of metabolic endotoxemia [17, 18], oral gavage of LPS in male WT mice increased serum levels of LPS 1.5-fold, 2 h after gavage treatment, *t*(8) = 16.96, *p* < 0.05 (one-tailed), *n* = 5/group, *η*^*2*^ = 0.34 [17, 18] (Fig. 1).

**Fig. 2:**
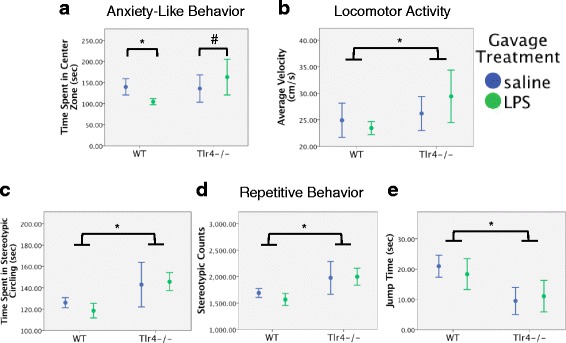
Effects of oral gavage of LPS on male WT and *Tlr4−/−* mice. Experiment 1: effects of oral gavage of LPS on open field test behavioral outcomes in male WT and *Tlr4−/−* mice. **a** LPS significantly decreased time in the center zone for male WT mice, but there was a slight trend toward increased time spent in the center zone for male *Tlr4−/−* mice. **b** Male *Tlr4−/−* mice had significantly increased average velocity, **c** time spent in stereotypic circling, and **d** number of stereotypic counts compared with male WT mice. **e** However, male WT mice had a significantly higher jump time compared to male *Tlr4−/−* mice. Error bars are 95% confidence intervals. *p < 0.05; #*p* = 0.107

2 × 2 (genotype × gavage treatment) univariate ANOVAs across all behavioral measures showed that oral gavage of LPS significantly increased anxiety-like behavior in WT mice but not *Tlr4−/−* mice (*n* = 7/group for WT mice and *n* = 4/group for *Tlr4−/−* mice). In comparison to vehicle treatment, LPS decreased time spent in the center for WT mice, *p* < 0.01 but not for *Tlr4−/−* mice (genotype × gavage treatment interaction *F*(1,18) = 14.051, *p* < 0.01). If anything, LPS tended to increase time spent in the center for *Tlr4−/−* mice, although this did not reach significance (*p* = 0.107, Fig. 2a). When collapsing across gavage treatment, WT mice had a higher jump time (*p* < 0.001, Fig. 2e), whereas *Tlr4−/−* mice had a higher average velocity (*p* < 0.01, Fig. 2b), spent more time in stereotypic circling (*p* < 0.001, Fig. 2c), and had a higher number of stereotypic counts (*p* < 0.001, Fig. 2d). Additional file 1: Table S1 lists ANOVA statistics for all measures across main effects and interactions, including locomotor parameters (ambulatory episodes, ambulatory counts, ambulatory time, ambulatory distance, resting time, and average velocity), anxiety-like behaviors (time spent in the center zone, zone entries, number of rears, and time spent rearing), and repetitive behaviors (time spent in stereotypic circling, number of stereotypic counts, jump counts, jump time, number of clockwise reversals, and number of counterclockwise reversals).

A 2 × 2 (genotype × gavage treatment) multivariate analysis of variance (MANOVA) was performed on all behavioral measures, wherein all main effects and interactions were non-significant (not reported). The observed power for the main effect of gavage treatment (power = 20.9%), the main effect of genotype (power = 46.1%), and the gavage treatment by genotype interaction effect (power = 43.2%) were all under the nominal 80% level. This suggests that this experimental cohort may have been underpowered for a factorial MANOVA.

**Fig. 3:**
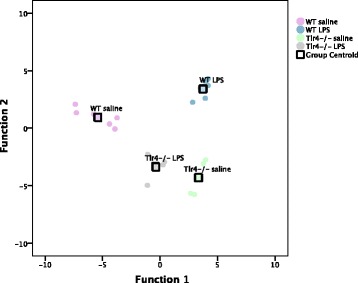
Canonical discriminant function plot for experiment 1. Experiment 1 discriminant function plot. Correlations between outcome variables and discriminant functions are listed in Table 1. Function 1 demonstrates an interaction between gavage treatment and genotype, mostly based on the differential effect of LPS on time spent in the center zone, time spent jumping, and jump counts in *Tlr4−/−* and WT mice, whereas function 2 separates groups based on genotype, largely driven by differences in number of stereotypic counts, time spent in stereotypic circling, and time spent in the center zone. Group centroids indicate the mean discriminant function value of each of the designated groups

Although MANOVA did not reveal significant effects of genotype or gavage treatment, discriminant analysis revealed the contribution of behavioral outcome variables to group separation by genotype and gavage treatment. When subjects were designated to four groups based on genotype and gavage treatment, discriminant analysis revealed three discriminant functions that maximize group separation based on genotype, gavage treatment, or the interaction between these two factors. Function 1 explains 60.0% of the variance, canonical *R*^2^ = 0.975; function 2 explains 35.6% of the variance, canonical *R*^2^ = 0.959; and function 3 explains 4.4% of the variance, canonical *R*^2^ = 0.765. Collectively, these discriminant functions significantly differentiated the treatment groups, *Λ* = 0.006, *Χ*^2^(42) = 77.077, *p* < 0.001. The structure matrix in Table 1 reveals the correlations between outcome variables and the discriminant functions. In the discriminant function plot (Fig. 3), function 1 demonstrates the opposing effects of gavage treatment on behavioral outcomes via the two genotypes, and the structure matrix shows this is predominantly driven by time spent in center zone (*r* = − 0.154), jump time (*r* = − 0.138), and jump counts (*r* = − 0.070) (Table 1). Function 2 separates groups by genotype, and this is driven predominantly by time spent in stereotypic circling (*r* = − 0.458), number of stereotypic counts (*r* = − 0.454), and time spent in the center zone (*r* = − 0.279) (Table 1). This suggests that while the genotypes are best distinguished on the basis of repetitive behaviors, gavage treatment affects anxiety-like and repetitive behavior differently in male WT and *Tlr4−/−* mice. Additional file 2: Table S2 demonstrates the results of a classification test to verify the validity of the discriminant functions plotted in Fig. 3. Using the original discriminant functions, 100% of the original grouped cases are correctly classified. These functions were further validated by a leave-one-out cross-validation procedure. Discriminant functions were re-computed with all subjects excluding one, and this procedure was repeated for all subjects. Across all analyses, 63.6% of cross-validated group cases were correctly classified, 38.6% above chance.

**Table 1:** Experiment 1 structure matrix (genotype by gavage treatment)

Measured outcome	Function		
	1	2	3
Jump counts	**−** *0.070*	0.070	− 0.026
Number of stereotypic counts	0.003	**−** *0.458*	0.186
Time spent in stereotypic circling	0.000	**−** *0.454*	*0.231*
Time spent jumping	**−** *0.138*	0.300	− 0.129
Ambulatory time	0.038	0.102	− 0.028
Time spent rearing	− 0.015	− 0.099	− 0.020
Number of ambulatory counts	0.025	0.069	0.045
Ambulatory distance	0.032	0.044	0.024
Time spent resting	− 0.017	− 0.024	− 0.014
Average velocity	− 0.031	− 0.216	*0.390*
Time spent in center zone	**−** *0.154*	**−** *0.279*	*0.333*
Number of clockwise reversals	− 0.029	0.019	0.171
Number of zone entries	− 0.020	− 0.098	0.153
Ambulatory episodes	0.019	− 0.025	0.120
Number of counterclockwise reversals	− 0.017	0.069	− 0.075

Function numbers match the order of the percentage of the variance explained by the respective functions. Each function maximizes separation between groups based on main effect of genotype, main effect of gavage treatment, or an interaction between these two factors, on the listed behaviors. Italicized numbers indicate the highest three correlations and therefore deemed most important for the discriminant function

### Experiment 2: sex differences in TLR4 agonism and antagonism on anxiety-like and repetitive behavior

In this experiment, we observed that oral gavage of LPS significantly increased anxiety behavior in both males and females, but a specific TLR4 antagonist, (+)-naloxone, had opposing effects on anxiety and repetitive behavior in males and females. Full factorial 2 (sex) × 2 (gavage treatment: LPS or saline) × 2 (i.p. injection: LPS-RS or saline) × 2 (i.p. injection: (+)-naloxone or saline) MANOVA was performed on the same dependent variables analyzed in experiment 1 (*n* = 8/group). There were significant main effects of sex (*F*(16,96) = 9.751, *p* < 0.001) and gavage treatment (*F*(16,96) = 2.111, *p* < 0.05), and a sex by (+)-naloxone interaction effect (*F*(16,96) = 2.176, *p* < 0.05). All remaining main effects and interactions, including effects of LPS-RS treatment, were non-significant (not reported). ANOVAs revealed these main effects and interactions across a number of behavioral parameters (Additional file 3: Table S3), of which subsequent planned contrasts indicated directionality.

**Fig. 4:**
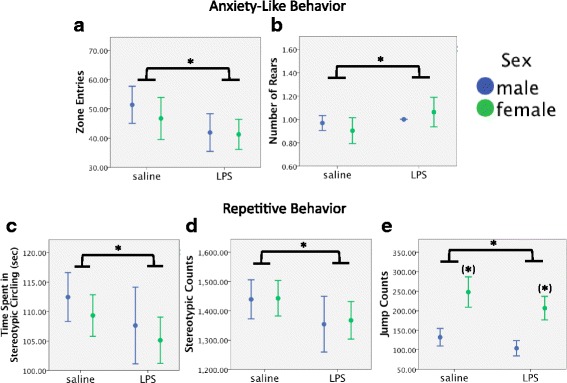
Effects of oral gavage of LPS on male and female WT mice. Experiment 2: effects of intraperitoneal injection of TLR4 antagonists and oral gavage of LPS on open field test behavioral outcomes in male and female WT mice. Data presented as sex by gavage treatment. LPS significantly **a** decreased zone entries and **b** increased the number of rears in males and females relative to saline-treated subjects. Furthermore, LPS significantly decreased the number of **c** time spent in stereotypic circling, **d** stereotypic counts, and **e** jump counts relative to saline-treated subjects. In addition, females had a higher jump count than males. Error bars are 95% confidence intervals. *p < 0.05; (*) significant main effect of sex, *p* < 0.05

These planned contrasts revealed that LPS suppressed repetitive and increased anxiety-like behaviors in males and females. Since no significant sex by LPS treatment effects were found in any of the ANOVAs or MANOVA, males and females were grouped together for subsequent analyses. LPS treatment decreased number of zone entries, *p* < 0.05 (Fig. 4a), and increased number of rears, *p* < 0.05 (Fig. 4b). Across both sexes, LPS treatment decreased time spent in stereotypic circling, *p* = 0.05 (Fig. 4c), stereotypic counts, *p* < 0.05 (Fig. 4d), and jump counts, *p* < 0.05 (Fig. 4e).

**Fig. 5:**
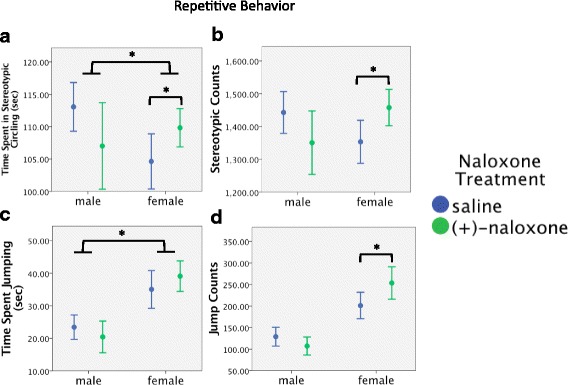
Effects of TLR4 antagonist (+)-naloxone treatment on male and female WT mice. Experiment 2: effects of intraperitoneal injection of TLR4 antagonists and oral gavage of LPS on open field test behavioral outcomes in male and female WT mice. Data presented as sex by (+)-naloxone treatment. **a** Overall, males spent significantly more time in stereotypic circling than females; however, (+)-naloxone significantly increased time in stereotypic circling in female mice. **b** In addition, (+)-naloxone significantly increased the number of stereotypic counts in female mice. For jumping behavior, (**c**) females jumped more than males, and **d** (+)-naloxone significantly increased jump counts in females. Error bars are 95% confidence intervals. *p < 0.05

When planned contrasts were run for the effects of (+)-naloxone in each sex separately, time spent in stereotypic circling (Fig. 5a), stereotypic counts (Fig. 5b) and jump counts (Fig. 5d) were significantly affected in females (*p* < 0.05, in each case) but not in males (*p* > 0.1, in each case).

**Fig. 6:**
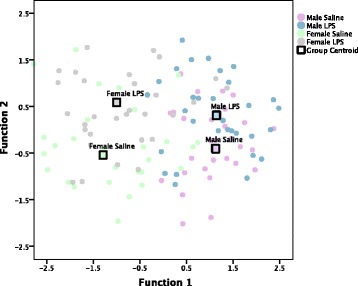
Canonical discriminant function plot for experiment 2 (sex × gavage treatment). Experiment 2 discriminant function plot (sex by gavage treatment). Correlations between outcome variables and discriminant functions are listed in Table 2. Function 1 separates groups based on sex, largely driven by differences in time spent jumping, jump time, and ambulatory time, whereas function 2 separates groups by gavage treatment, mostly driven by number of rears, zone entries, and stereotypic counts. LPS affects males and females in a similar fashion across discriminant function 2. Group centroids indicate the mean discriminant function value of each of the designated groups

Discriminant analysis confirms that LPS increased anxiety behavior similarly in males and females, while (+)-naloxone had different effects in males and females. As for gavage treatment, discriminant analysis revealed three discriminant functions on data from subjects grouped by sex and gavage treatment. Function 1 explains 80.6% of the variance, canonical *R*^2^ = 0.758; function 2 explains 13.8% of the variance, canonical *R*^2^ = 0.433; and function 3 explains 5.6% of the variance, canonical *R*^2^ = 0.292. Collectively, these discriminant functions significantly differentiated the treatment groups, *Λ* = 0.317, *Χ*^2^(45) = 133.937, *p* < 0.001. The discriminant function plot (Fig. 6) shows that function 1 separates groups based on sex, and the structure matrix (Table 2) reveals this is predominantly driven by sex differences in jump counts (*r* = − 0.616), jump time (*r* = − 0.508), and ambulatory time (*r* = − 0.392). Function 2 separates groups based on gavage treatment, and LPS appears to affect males and females in a similar fashion along outcome variables, predominantly number of rears (*r* = 0.453), zone entries (*r* = − 0.429), and stereotypic counts (*r* = − 0.400). Additional file 4: Table S4 displays results of the original grouping and leave-one-out classification tests. 64.6% of original grouped cases were correctly classified, and 45.7% of cross-validated grouped cases were correctly classified, 20.7% above chance. (The same group of behavioral measures used in experiment 1 was also used in discriminant analyses performed for experiment 2. Only behavioral measures that differentiate the discriminant functions are listed in the structure matrix.)

**Table 2:** Experiment 2 structure matrix (sex by gavage treatment)

Measured outcome	Function		
	1	2	3
Jump counts	*− 0.616*	− 0.354	0.124
Jump time	*− 0.508*	− 0.270	0.139
Ambulatory time	*− 0.392*	− 0.193	− 0.108
Ambulatory counts	− 0.368	− 0.283	− 0.116
Ambulatory distance	− 0.345	− 0.270	− 0.060
Resting time	0.344	0.295	0.014
Ambulatory episodes	− 0.330	− 0.204	− 0.120
Average velocity	− 0.171	− 0.161	0.160
Number of rears	0.018	*0.453*	*0.276*
Zone entries	0.057	*− 0.429*	*0.338*
Stereotypic counts	− 0.028	*− 0.400*	0.189
Time spent in stereotypic circling	0.086	− 0.372	0.154
Time spent rearing	0.144	0.285	0.223
Counterclockwise reversals	− 0.114	− 0.252	− 0.055
Time spent in center zone	0.024	− 0.094	*0.463*
Clockwise reversals	− 0.042	− 0.080	− 0.081

Function numbers match the order of the percentage of the variance explained by the respective functions. Each function maximizes separation between groups based on main effect of sex, main effect of gavage treatment, or an interaction between these two factors, on the listed behaviors. Italicized numbers indicate the highest three correlations and therefore deemed most important for the discriminant function

**Fig. 7:**
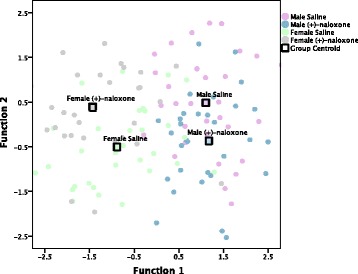
Canonical discriminant function plot for experiment 2 (sex × (+)-naloxone treatment). Experiment 2 discriminant function plot (sex by (+)-naloxone treatment). Correlations between outcome variables and discriminant functions are listed in Table 3. Function 1 separates groups based on sex, largely driven by differences in time spent jumping, jump time, and ambulatory time, whereas function 2 demonstrates an interaction between sex and (+)-naloxone treatment, mostly based on the sexually differential effect of (+)-naloxone on stereotypic counts, ambulatory episodes, and time spent in stereotypic circling. Group centroids indicate the mean discriminant function value of each of the designated groups

Discriminant analysis also indicates that (+)-naloxone affected repetitive and locomotor behaviors differently in males and females, revealing three discriminant functions (Fig. 7, Table 3). Rear count is automatically excluded from the discriminant analysis, based on its lack of contribution to the discriminant functions. Function 1 explains 81.5% of the variance, canonical *R*^2^ = 0.764, Function 2 explains 11.8% of the variance, canonical *R*^2^ = 0.410, and function 3 explains 6.7% of the variance, canonical *R*^2^ = 0.322. Collectively, these discriminant functions significantly differentiated the treatment groups, *Λ* = 0.311, *Χ*^2^(45) = 136.022, *p* < 0.001. As shown in the discriminant analysis where subjects are grouped by sex and gavage treatment, the discriminant function plot shows that function 1 separates groups based on sex and the structure matrix reveals this is predominantly driven by sex differences in jump counts (*r* = − 0.623), jump time (*r* = − 0.496), and ambulatory time (*r* = − 0.416). Function 2 separates groups based on (+)-naloxone treatment, and (+)-naloxone appears to affect males and females differently along outcome variables, predominantly stereotypic counts (*r* = 0.545), ambulatory episodes (*r* = 0.526), and time spent in stereotypic circling (*r* = 0.523). Additional file 5: Table S5 displays results of the original grouping and leave-one-out classification tests. 65.4% of original grouped cases were correctly classified, and 48.0% of cross-validated grouped cases were correctly classified, 23.0% above chance.

**Table 3:** Experiment 2 structure matrix (sex by (+)-naloxone treatment)

Measured outcome	Function		
	1	2	3
Jump counts	*− 0.623*	0.319	− 0.057
Jump time	*− 0.496*	0.123	0.122
Ambulatory time	*− 0.416*	0.414	− 0.134
Counterclockwise reversals	− 0.106	0.086	0.090
Stereotypic counts	− 0.046	*0.545*	− 0.008
Ambulatory episodes	− 0.363	*0.526*	− 0.140
Time spent in stereotypic circling	0.070	*0.523*	0.044
Time spent resting	0.367	− 0.485	0.068
Ambulatory counts	− 0.390	0.442	− 0.166
Ambulatory distance	− 0.369	0.439	− 0.188
Average velocity	− 0.180	0.397	*0.345*
Number of rears	0.020	− 0.285	0.174
Time spent rearing	0.144	− 0.086	*0.327*
Clockwise reversals	− 0.042	0.181	*0.197*
Zone entries	0.058	0.075	− 0.138
Time spent in center zone	0.022	− 0.061	− 0.104

Function numbers match the order of the percentage of the variance explained by the respective functions. Each function maximizes separation between groups based on main effect of sex, main effect of (+)-naloxone treatment, or an interaction between these two factors, on the listed behaviors. Italicized numbers indicate the highest three correlations and therefore deemed most important for the discriminant function

**Fig. 8:**
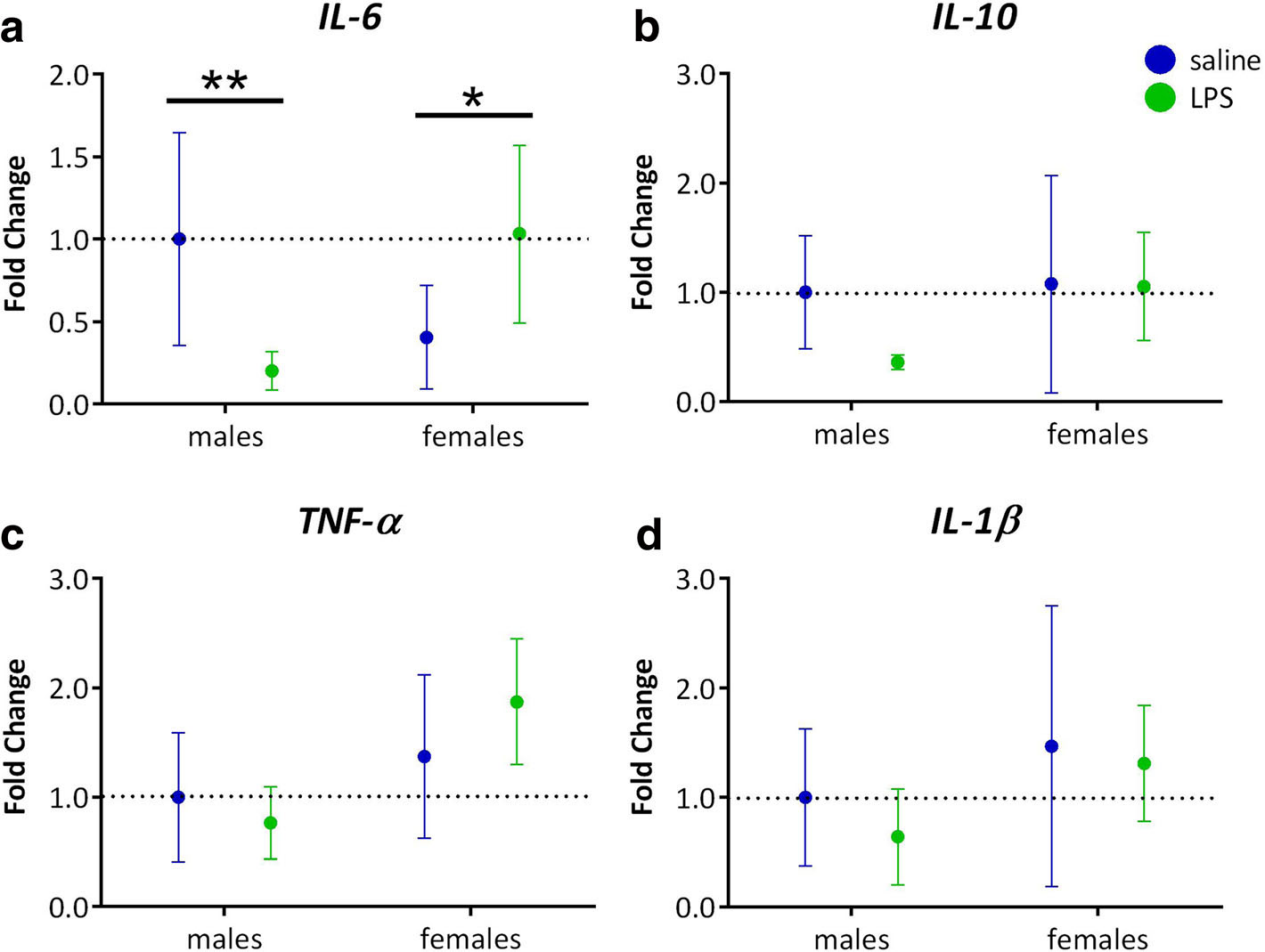
Effects of oral gavage of LPS on gut cytokine expression in female and male mice. **a** Expression levels of *IL-6* showed a sex-dependent effect, with LPS causing a reduction of *IL-6* in males and an increase in females. **b**–**d** However, the expression of *IL-10*, *TNF-α*, and *IL-1β* did not depend on sex or experimental treatment. Data are expressed relative to levels of saline-treated males. Error bars are 95% confidence intervals. *p < 0.05; **p < 0.01

Since neither TLR4 antagonist blocked the specific effects of LPS, we sought to identify the systemic and intestinal inflammatory effects of the oral LPS treatment in WT mice not treated with TLR4 antagonists. Gavage treatment resulted in non-significant elevation of serum endotoxin levels in male subjects in a meta-analysis across experiments 1 and 2 (*p* > 0.05) (Additional file 6: Figure S1), and there were no significant main effects of sex or gavage treatment, or interactions between sex and gavage treatment, on serum LPS levels in experiment 2 (data not shown). In addition, cytokine Luminex was performed on serum samples from experiment 2. There were no significant effects of gavage treatment on serum levels of TNF-α, IL-1β, IL-6, or IL-10 (data not shown). However, oral gavage of LPS did modulate IL-6 expression levels in intestinal tissue in a sexually differentiated manner (sex-by-treatment interaction *F*(1,21) = 12.38, *p* = 0.002), increasing IL-6 expression in females (*p* = 0.04) while decreasing it in males (*p* = 0.009, Fig. 8a). IL-10, TNF-α, and IL-1β expression levels were not significantly altered by the oral LPS treatment (Fig. 8b–d).

### Meta-analyses across experiments 1 and 2

**Fig. 9:**
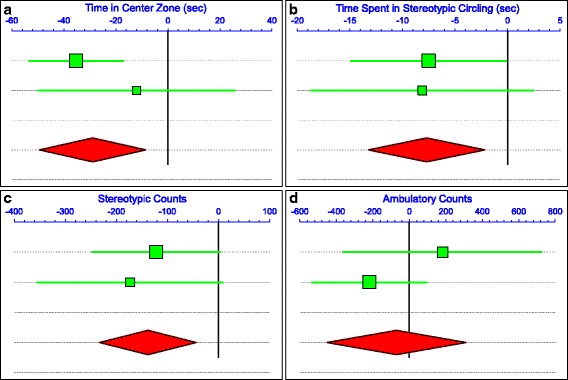
Meta-analyses of behavior in WT males of experiments 1 and 2. Forest plots of meta-analyses of **a** time spent in center zone, **b** time spent in stereotypic circling, **c** stereotypic counts, and **d** ambulatory counts measured in experiment 1 (top green bar) and experiment 2 (bottom green bar). The result of the meta-analysis is indicated by the red diamond. The width of the green bars and the red diamond indicate the range of the 95% confidence intervals for each, with the center representing the mean. WT males not treated with TLR4 antagonists were used for these analyses (total *n* = 15/group). See Additional file 18: Table S7 for statistics

A random effects meta-analysis was performed across experimental cohorts in order to obtain more general estimates of the effects of oral gavage of LPS on behavior. As both experiments used WT males, all WT males from experiment 1 and WT males not treated with TLR4 antagonists in experiment 2 were used for the meta-analyses (total *n* = 15/group). Across experiments 1 and 2, there were significant effects of LPS on time spent in the center zone (*p* < 0.01), time spent in stereotypic circling (*p* < 0.01), and stereotypic counts (*p* < 0.01) (Fig. 9). There were non-significant effects of LPS on parameters of locomotion, including ambulatory counts (*p* > 0.1), ambulatory episodes (*p* > 0.10), ambulatory time (*p* > 0.1), ambulatory distance (*p* > 0.10), resting time (*p* > 0.10), and average velocity (*p* > 0.10). All other behavioral parameters were also non-significant (Additional file 7: Figure S2; Additional file 8: Figure S3; Additional file 9: Figure S4; Additional file 10: Figure S5; Additional file 11: Figure S6; Additional file 12: Figure S7; Additional file 13: Figure S8; Additional file 14: Figure S9; Additional file 15: Figure S10; Additional file 16: Figure S11; Additional file 17: Figure S12 and Additional file 18: Table S7).

## Discussion

Here, we observe that gut-derived LPS elicits various aspects of the canonical sickness behavior response, with the exception of lethargy. In our first experiment, LPS increased anxiety-like behavior in male WT mice whereas no such effect was found in male *Tlr4−/−* mice. In the second experiment, LPS similarly increased anxiety-like behaviors in WT males and females. Neither TLR4 antagonist ((+)-naloxone nor LPS-RS) blocked the effects of gavage treatment. However, (+)-naloxone, a TLR4/TRIF specific antagonist, which does not interact with opioid receptors [31, 32], affected behavior differently in males and females. Furthermore, LPS-RS did not significantly alter behavior, suggesting that the MyD88 pathway may not be involved in anxiety-like and repetitive behaviors generated by gut-derived endotoxin. With the presented data, we offer oral administration of LPS as a model of gut dysbiosis that may result from overgrowth of pathogenic gram-negative bacteria in gut microbiota.

While LPS increased anxiety behavior 2 h after treatment, it did not increase lethargy (indexed as hypolocomotion), as seen 2–6 h after systemic injections of LPS. Importantly, oral gavage of LPS induced similar increases in anxiety-like behavior to those observed after direct injection [44–54]. This suggests that oral gavage of LPS specifically induced anxiety-like behavior without inducing a generalized sickness response.

In experiment 1, an oral gavage of LPS increased anxiety-like behaviors in male WT mice, as measured by decreased time spent in the center zone of the open field test. In experiment 2, the oral gavage of LPS did not strongly affect time spent in the center zone in subject mice. This may be a result of increased anxiety stemming from the additional manipulations (e.g., intraperitoneal injection) in this experiment as both gavage and injections can increase anxiety [30, 55] or from behavioral variability across experimental cohorts. Nevertheless, multivariate analyses from experiment 2 indicate that gut-derived LPS produced a syndrome of behavioral alterations that includes increases in anxiety-like behaviors (increased incidence of vertical stretch posture and decreased zone entries) and decreases in repetitive behaviors (decreased jump time and jump counts), albeit along a slightly different combination of measures from that found in experiment 1. In support of the conclusion from the multivariate analyses that LPS affects anxiety-like behaviors in both experiments, meta-analysis of LPS effects in WT males indicates that the observed reduction in time spent in the center zone, a highly used index of anxiety-like behavior [56, 57], is similar to the reported range of reduced time spent in the center zone for male mice injected intraperitoneally or intravenously with LPS (20 to 60 s difference per 5 min segment) [44–54]. Overall, these data demonstrate the utility of multivariate analyses to highlight similar behavioral effects across differing contexts.

Our data indicate that the behavioral effects of gut-derived LPS are mediated through TLR4. Oral administration of LPS significantly increased anxiety-like behavior in WT mice but not in *Tlr4−/−* mice. If anything, there was a trend toward LPS increasing time spent in the center zone in *Tlr4−/−* mice, suggesting that LPS may interact with other innate immune receptors to decrease anxiety. TLR4 antagonists, however, did not directly block the effects of LPS gavage on behavior. It is unlikely that this is due to ineffective dosage, as we chose dosages of antagonists based on the literature [36–38, 58], and (+)-naloxone affected behavior regardless of LPS treatment in this study. Measurement of cytokines suggests that LPS acted primarily at the level of the gut, as we did not find elevation of inflammatory markers in serum but did find a significant elevation of IL-6 expression in the gut. If so, it may be that the TLR4 antagonists did not intervene effectively at the site of action of the LPS. Orally administered LPS likely interacts with TLR4 present on the apical surface of intestinal epithelial cells. It is plausible that our antagonists, when injected intraperitoneally, do not have sufficient access to these receptors.

Our data suggest that there may be sex differences in constitutive TLR4 activity and its downstream effects on locomotor and repetitive behaviors. The antagonist (+)-naloxone, which blocks the TLR4/TRIF signaling pathway, increased stereotypic circling time and ambulatory episodes in females while decreasing these behaviors in males, regardless of gavage treatment. This suggests that the TLR4/TRIF pathway differently modulates these behaviors in males and females. This is in line with literature that shows (+)-naloxone more effectively blocks TLR4-modulated nociception in female than in male rats [59]. In our study, oral LPS treatment increased intestinal IL-6 expression in females and suppressed it in males. As IL-6 expression depends on the TLR4/TRIF pathway [60], it is possible that sex differences in this pathway contributed to sex differences in LPS effects on IL-6 observed in this study.

Our data also demonstrate that TLR4 activation may suppress repetitive behaviors. Genetic deletion of TLR4 in males and blockade of TLR4 signaling (with (+)-naloxone) in females both increase stereotypic circling. These effects may possibly be driven by the suppression of allergic-type (Th2-driven) immune profiles by TLR4, as *Tlr4−/−* mice are reported to show enhanced allergic responses [61, 62]. In line with this prediction, a number of studies demonstrate that allergic-type immune profiles increase repetitive behaviors [63–70]. It is notable that the TLR4 antagonist (+)-naloxone decreased stereotypic circling in males, while enhancing it in females. There are documented sex differences in cytokine responses to TLR4 activation [59, 71]. Our data further suggest potential sex differences in the TLR4/TRIF pathway in males and females, and these differences may contribute to the sex difference we observed in stereotypic circling among saline-treated mice. Alignment of the effects of TLR4 genetic mutation in males and effects of (+)-naloxone in females, and contrary effects of (+)-naloxone in males, suggests that TLR4 may play the same role in males and females, but the underlying signaling pathways may differ between the sexes.

## Conclusion

In spite of the ubiquitous nature of LPS in the gut lumen, this is the first study to demonstrate that gut-derived LPS can initiate behavioral aspects of the sickness response. Our results suggest that an increased intestinal load of LPS similarly increases anxiety-like behavior and suppresses repetitive behavior in males and females. However, to the extent this is mediated through TLR4 activation, this may occur via differing mechanisms. Furthermore, different actions of the TLR4/TRIF pathway may drive baseline differences in repetitive behaviors in males and females.

## Additional files

Additional file 1: Table S1. Title: Independent ANOVAs from Experiment 1 suggest outcome variables that contribute to group differences highlighted by Pillai’s trace. Legend: Individual ANOVAs on outcome variables measured in Experiment 1. Significant results are boldfaced. F values are indicated in the “F” column, *p* values are indicated in the “Sig.” column and effect sizes (partial eta squared) are indicated in the “Partial η^2” column. For each ANOVA, hypothesis degrees of freedom is 1 and error degrees of freedom is 18. (DOCX 16 kb)

Additional file 2: Table S2. Title: Original classification and cross-validation of discriminant functions for Experiment 1. Legend: Validation of discriminant functions for Experiment 1 by original case classification and leave-one-out cross validation. 100% of the original grouped cases are correctly classified by the discriminant functions. In the leave-one-out cross-validation test, the discriminant functions are recalculated excluding one case, and all cases are recalculated. This algorithm is repeated for the exclusion of each case. In the leave-one-out test, 63.6% of cross-validated grouped cases were correctly classified. (DOCX 14 kb)

Additional file 3: Table S3. Title: Independent ANOVAs from Experiment 2 suggest outcome variables that contribute to group differences highlighted by Pillai’s trace. Legend: Individual ANOVAs on outcome variables measured in Experiment 2. Significant results are boldfaced. Trends are italicized. F values are indicated in the “F” column, p values are indicated in the “Sig.” column and effect sizes (partial eta squared) are indicated in the “Partial η^2” column. For each ANOVA, hypothesis degrees of freedom is 1 and error degrees of freedom is 111. (DOCX 39 kb)

Additional file 4: Table S4. Title: Original classification and cross-validation of discriminant functions for Experiment 2 for cases grouped by gavage treatment and sex. Legend: Validation of discriminant functions for Experiment 2, for cases grouped by gavage treatment and sex, by original case classification and leave-one-out cross validation. 64.6% of the original grouped cases are correctly classified by the discriminant functions. In the leave-one-out cross-validation test, the discriminant functions are recalculated excluding one case, and all cases are recalculated. This algorithm is repeated for the exclusion of each case. In the leave-one-out test, 45.7% of cross-validated grouped cases were correctly classified. (DOCX 14 kb)

Additional file 5: Table S5. Title: Original classification and cross-validation of discriminant functions for Experiment 1. Legend: Validation of discriminant functions for Experiment 2, for cases grouped by (+)-naloxone treatment and sex, by original case classification and leave-one-out cross validation. 65.4% of the original grouped cases are correctly classified by the discriminant functions. In the leave-one-out cross-validation test, the discriminant functions are recalculated excluding one case, and all cases are recalculated. This algorithm is repeated for the exclusion of each case. In the leave-one-out test, 48.0% of cross-validated grouped cases were correctly classified. (DOCX 14 kb)

Additional file 6: Figure S1. Title: Meta-analysis of serum endotoxin levels in WT males of Experiments 1 and 2. Legend: Forest plot of difference in serum endotoxin levels between male WT subjects gavaged with saline or LPS, measured from Experiment 1 (top green bar) and Experiment 2 (bottom green bar). The result of the meta-analysis is indicated by the red diamond. The width of the green bars and the red diamond indicate the range of the 95% confidence intervals for each, with the center representing the mean. WT males not treated with TLR4 antagonists were used for these analyses (total *n* = 15/group). (PPTX 45 kb)

Additional file 7: Figure S2. Title: Meta-analysis of ambulatory episodes in WT males of Experiments 1 and 2. Legend: Forest plot of difference in ambulatory episodes between male WT subjects gavaged with saline or LPS, measured from Experiment 1 (top green bar) and Experiment 2 (bottom green bar). The result of the meta-analysis is indicated by the red diamond. The width of the green bars and the red diamond indicate the range of the 95% confidence intervals for each, with the center representing the mean. WT males not treated with TLR4 antagonists were used for these analyses (total *n* = 15/group). See Additional file 18: Table S7 for statistics. (PPTX 45 kb)

Additional file 8: Figure S3. Title: Meta-analysis of ambulatory time in WT males of Experiments 1 and 2. Legend: Forest plot of difference in ambulatory time between male WT subjects gavaged with saline or LPS, measured from Experiment 1 (top green bar) and Experiment 2 (bottom green bar). The result of the meta-analysis is indicated by the red diamond. The width of the green bars and the red diamond indicate the range of the 95% confidence intervals for each, with the center representing the mean. WT males not treated with TLR4 antagonists were used for these analyses (total *n* = 15/group). See Additional file 18: Table S7 for statistics. (PPTX 45 kb)

Additional file 9: Figure S4. Meta-analysis of ambulatory distance in WT males of Experiments 1 and 2. Legend: Forest plot of difference in ambulatory distance between male WT subjects gavaged with saline or LPS, measured from Experiment 1 (top green bar) and Experiment 2 (bottom green bar). The result of the meta-analysis is indicated by the red diamond. The width of the green bars and the red diamond indicate the range of the 95% confidence intervals for each, with the center representing the mean. WT males not treated with TLR4 antagonists were used for these analyses (total *n* = 15/group). See Additional file 18: Table S7 for statistics. (PPTX 45 kb)

Additional file 10: Figure S5. Meta-analysis of resting time in WT males of Experiments 1 and 2. Legend: Forest plot of difference in resting time between male WT subjects gavaged with saline or LPS, measured from Experiment 1 (top green bar) and Experiment 2 (bottom green bar). The result of the meta-analysis is indicated by the red diamond. The width of the green bars and the red diamond indicate the range of the 95% confidence intervals for each, with the center representing the mean. WT males not treated with TLR4 antagonists were used for these analyses (total *n* = 15/group). See Additional file 18: Table S7 for statistics. (PPTX 45 kb)

Additional file 11: Figure S6. Meta-analysis of average velocity in WT males of Experiments 1 and 2. Legend: Forest plot of difference in average velocity between male WT subjects gavaged with saline or LPS, measured from Experiment 1 (top green bar) and Experiment 2 (bottom green bar). The result of the meta-analysis is indicated by the red diamond. The width of the green bars and the red diamond indicate the range of the 95% confidence intervals for each, with the center representing the mean. WT males not treated with TLR4 antagonists were used for these analyses (total *n* = 15/group). See Additional file 18: Table S7 for statistics. (PPTX 45 kb)

Additional file 12: Figure S7. Meta-analysis of zone entries in WT males of Experiments 1 and 2. Legend: Forest plot of difference in zone entries between male WT subjects gavaged with saline or LPS, measured from Experiment 1 (top green bar) and Experiment 2 (bottom green bar). The result of the meta-analysis is indicated by the red diamond. The width of the green bars and the red diamond indicate the range of the 95% confidence intervals for each, with the center representing the mean. WT males not treated with TLR4 antagonists were used for these analyses (total *n* = 15/group). See Additional file 18: Table S7 for statistics. (PPTX 45 kb)

Additional file 13: Figure S8. Meta-analysis of stretch posture in WT males of Experiments 1 and 2. Legend: Forest plot of difference in time in stretch posture between male WT subjects gavaged with saline or LPS, measured from Experiment 1 (top green bar) and Experiment 2 (bottom green bar). The result of the meta-analysis is indicated by the red diamond. The width of the green bars and the red diamond indicate the range of the 95% confidence intervals for each, with the center representing the mean. WT males not treated with TLR4 antagonists were used for these analyses (total *n* = 15/group). See Additional file 18: Table S7 for statistics. (PPTX 45 kb)

Additional file 14: Figure S9. Meta-analysis of jump counts in WT males of Experiments 1 and 2. Legend: Forest plot of difference in jump counts between male WT subjects gavaged with saline or LPS, measured from Experiment 1 (top green bar) and Experiment 2 (bottom green bar). The result of the meta-analysis is indicated by the red diamond. The width of the green bars and the red diamond indicate the range of the 95% confidence intervals for each, with the center representing the mean. WT males not treated with TLR4 antagonists were used for these analyses (total *n* = 15/group). See Additional file 18: Table S7 for statistics. (PPTX 45 kb)

Additional file 15: Figure S10. Meta-analysis of jump time in WT males of Experiments 1 and 2. Legend: Forest plot of difference in jump time between male WT subjects gavaged with saline or LPS, measured from Experiment 1 (top green bar) and Experiment 2 (bottom green bar). The result of the meta-analysis is indicated by the red diamond. The width of the green bars and the red diamond indicate the range of the 95% confidence intervals for each, with the center representing the mean. WT males not treated with TLR4 antagonists were used for these analyses (total n = 15/group). See Additional file 18: Table S7 for statistics (PPTX 45 kb)

Additional file 16: Figure S11. Meta-analysis of clockwise reversals in WT males of Experiments 1 and 2. Legend: Forest plot of difference in clockwise reversals between male WT subjects gavaged with saline or LPS, measured from Experiment 1 (top green bar) and Experiment 2 (bottom green bar). The result of the meta-analysis is indicated by the red diamond. The width of the green bars and the red diamond indicate the range of the 95% confidence intervals for each, with the center representing the mean. WT males not treated with TLR4 antagonists were used for these analyses (total n = 15/group). See Additional file 18: Table S7 for statistics. (PPTX 45 kb)

Additional file 17: Figure S12. Meta-analysis of counter-clockwise reversals in WT males of Experiments 1 and 2. Legend: Forest plot of difference in counter-clockwise reversals between male WT subjects gavaged with saline or LPS, measured from Experiment 1 (top green bar) and Experiment 2 (bottom green bar). The result of the meta-analysis is indicated by the red diamond. The width of the green bars and the red diamond indicate the range of the 95% confidence intervals for each, with the center representing the mean. WT males not treated with TLR4 antagonists were used for these analyses (total n = 15/group). See Additional file 18: Table S7 for statistics. (PPTX 45 kb)

Additional file 18: Table S6. Meta-analysis of behavioral outcomes for WT males not treated with TLR4 antagonists (n = 15/group). Legend: 95% confidence interval values for Experiment 1, Experiment 2, and the meta-analysis. UL = Upper Limit. LL = Lower Limit. (DOCX 17 kb)

## Abbreviations

LPS: Lipopolysaccharide
MANOVA: Multivariate analysis of variance
MyD88: Myeloid differentiation primary response gene 88
TLR4: Toll-like receptor 4
TRIF: TIR-domain-containing adapter-inducing interferon-beta
WT: Wild-type
