# Supplementary material for: Effects of gut-derived endotoxin on anxiety-like and repetitive behaviors in male and female mice
Source: Biol Sex Differ. 2018 Jan 19;9:7. doi: 10.1186/s13293-018-0166-x (PMC5775597; doi:10.1186/s13293-018-0166-x)
Supplement: Supplementary file 1 — Title: Independent ANOVAs from Experiment 1 suggest outcome variables that contribute to group differences highlighted by Pillai’s trace. Legend: Individual ANOVAs on outcome variables measured in Experiment 1. Significant results are boldfaced. F values are indicated in the “F” column, p values are indicated in the “Sig.” column and effect sizes (partial eta squared) are indicated in the “Partial η^2” column. For each ANOVA, hypothesis degrees of freedom is 1 and error degrees of freedom is 18. (DOCX 16 kb) [file 13293_2018_166_MOESM1_ESM.docx]

Additional file 1: Table S1: Title: Independent ANOVAs from Experiment 1 suggest outcome variables that contribute to group differences highlighted by Pillai’s trace.

| Source | Dependent Variable | F | Sig. | Partial η^2 |
| --- | --- | --- | --- | --- |
| Gavage Treatment | Time in Center Zone | 0.25 | 0.623 | 0.014 |
|  | Number of Center Zone Entries | 0.040 | 0.843 | 0.002 |
|  | Time Spent in Stereotypic Circling | 0.489 | 0.493 | 0.026 |
|  | Number of Clockwise Reversals | 0.459 | 0.507 | 0.025 |
|  | Time Spent in Vertical Stretch Posture | 0.32 | 0.579 | 0.017 |
|  | Jump Counts | 0.078 | 0.783 | 0.004 |
|  | Number of Counterclockwise Reversals | 0.032 | 0.86 | 0.002 |
|  | Time Spent Jumping | 0.08 | 0.781 | 0.004 |
|  | Incidence of Vertical Stretch Posture | . | . | . |
|  | Ambulatory Episodes | 0.327 | 0.574 | 0.018 |
|  | Ambulatory Counts | 0.393 | 0.539 | 0.021 |
|  | Ambulatory Distance | 0.229 | 0.638 | 0.013 |
| Genotype | **Time in Center Zone** | **10.742** | **0.004** | **0.374** |
|  | Number of Center Zone Entries | 1.857 | 0.190 | 0.094 |
|  | **Time Spent in Stereotypic Circling** | **40.597** | **0** | **0.693** |
|  | Number of Clockwise Reversals | 0.068 | 0.797 | 0.004 |
|  | Time Spent in Vertical Stretch Posture | 1.564 | 0.227 | 0.08 |
|  | Jump Counts | 1.813 | 0.195 | 0.091 |
|  | Number of Counterclockwise Reversals | 1.197 | 0.288 | 0.062 |
|  | **Time Spent Jumping** | **24.089** | **0** | **0.572** |
|  | Incidence of Vertical Stretch Posture | . | . | . |
|  | Ambulatory Episodes | 0.302 | 0.589 | 0.017 |
|  | Ambulatory Counts | 0.591 | 0.452 | 0.032 |
|  | Ambulatory Distance | 0.168 | 0.687 | 0.009 |
| Gavage Treatment | **Time in Center Zone** | **14.051** | **0.001** | **0.438** |
| by Genotype | Numbe of Center Zone Entries | 0.865 | 0.365 | 0.046 |
|  | Time Spent in Stereotypic Circling | 2.187 | 0.156 | 0.108 |
|  | Number of Clockwise Reversals | 0.86 | 0.366 | 0.046 |
|  | Time Spent in Vertical Stretch Posture | 0.106 | 0.749 | 0.006 |
|  | Jump Counts | 0.682 | 0.42 | 0.036 |
|  | Number of Counterclockwise Reversals | 0.01 | 0.922 | 0.001 |
|  | Time Spent Jumping | 1.231 | 0.282 | 0.064 |
|  | Incidence of Vertical Stretch Posture | . | . | . |
|  | Ambulatory Episodes | 0.018 | 0.894 | 0.001 |
|  | Ambulatory Counts | 0.121 | 0.732 | 0.007 |
|  | Ambulatory Distance | 0.216 | 0.647 | 0.012 |

Legend: Individual ANOVAs on outcome variables measured in Experiment 1. Significant results are boldfaced. F values are indicated in the “F” column, p values are indicated in the "Sig." column and effect sizes (partial eta squared) are indicated in the "Partial η^2" column. For each ANOVA, hypothesis degrees of freedom is 1 and error degrees of freedom is 18.
