# Supplementary material for: Effects of gut-derived endotoxin on anxiety-like and repetitive behaviors in male and female mice
Source: Biol Sex Differ. 2018 Jan 19;9:7. doi: 10.1186/s13293-018-0166-x (PMC5775597; doi:10.1186/s13293-018-0166-x)
Supplement: Supplementary file 2 — Title: Original classification and cross-validation of discriminant functions for Experiment 1. Legend: Validation of discriminant functions for Experiment 1 by original case classification and leave-one-out cross validation. 100% of the original grouped cases are correctly classified by the discriminant functions. In the leave-one-out cross-validation test, the discriminant functions are recalculated excluding one case, and all cases are recalculated. This algorithm is repeated for the exclusion of each case. In the leave-one-out test, 63.6% of cross-validated grouped cases were correctly classified. (DOCX 14 kb) [file 13293_2018_166_MOESM2_ESM.docx]

Additional file 2: Table S2: Title: Original classification and cross-validation of discriminant functions for Experiment 1.

| **Classification Results^a,c^** | | | | | | | |
| --- | --- | --- | --- | --- | --- | --- | --- |
|  |  | Complete Label Code | Predicted Group Membership | | | | Total |
|  |  |  | WT saline | WT LPS | Tlr4-‎/- saline | Tlr4-‎/- LPS |  |
| Original | Count | WT saline | 7 | 0 | 0 | 0 | 7 |
|  |  | WT LPS | 0 | 7 | 0 | 0 | 7 |
|  |  | Tlr4-‎/- saline | 0 | 0 | 4 | 0 | 4 |
|  |  | Tlr4-‎/- LPS | 0 | 0 | 0 | 4 | 4 |
|  | % | WT saline | 100.0 | .0 | .0 | .0 | 100.0 |
|  |  | WT LPS | .0 | 100.0 | .0 | .0 | 100.0 |
|  |  | Tlr4-‎/- saline | .0 | .0 | 100.0 | .0 | 100.0 |
|  |  | Tlr4-‎/- LPS | .0 | .0 | .0 | 100.0 | 100.0 |
| Cross-validated^b^ | Count | WT saline | 4 | 0 | 0 | 3 | 7 |
|  |  | WT LPS | 0 | 6 | 0 | 1 | 7 |
|  |  | Tlr4-‎/- saline | 0 | 0 | 2 | 2 | 4 |
|  |  | Tlr4-‎/- LPS | 1 | 0 | 1 | 2 | 4 |
|  | % | WT saline | 57.1 | .0 | .0 | 42.9 | 100.0 |
|  |  | WT LPS | .0 | 85.7 | .0 | 14.3 | 100.0 |
|  |  | Tlr4-‎/- saline | .0 | .0 | 50.0 | 50.0 | 100.0 |
|  |  | Tlr4-‎/- LPS | 25.0 | .0 | 25.0 | 50.0 | 100.0 |

Legend: Validation of discriminant functions for Experiment 1 by original case classification and leave-one-out cross validation. 100% of the original grouped cases are correctly classified by the discriminant functions. In the leave-one-out cross-validation test, the discriminant functions are recalculated excluding one case, and all cases are recalculated. This algorithm is repeated for the exclusion of each case. In the leave-one-out test, 63.6% of cross-validated grouped cases were correctly classified.
