# Supplementary material for: Effects of gut-derived endotoxin on anxiety-like and repetitive behaviors in male and female mice
Source: Biol Sex Differ. 2018 Jan 19;9:7. doi: 10.1186/s13293-018-0166-x (PMC5775597; doi:10.1186/s13293-018-0166-x)
Supplement: Supplementary file 3 — Title: Independent ANOVAs from Experiment 2 suggest outcome variables that contribute to group differences highlighted by Pillai’s trace. Legend: Individual ANOVAs on outcome variables measured in Experiment 2. Significant results are boldfaced. Trends are italicized. F values are indicated in the “F” column, p values are indicated in the “Sig.” column and effect sizes (partial eta squared) are indicated in the “Partial η^2” column. For each ANOVA, hypothesis degrees of freedom is 1 and error degrees of freedom is 111. (DOCX 39 kb) [file 13293_2018_166_MOESM3_ESM.docx]

Additional file 3: Table S6: Title: Independent ANOVAs from Experiment 2 suggest outcome variables that contribute to group differences highlighted by Pillai’s trace.

| Source | Dependent Variable | F | Sig. | Partial Eta Squared |
| --- | --- | --- | --- | --- |
| Sex | **Jump Counts** | **62.354** | **0** | **0.36** |
|  | **Jump Time** | **39.281** | **0** | **0.261** |
|  | **Ambulatory Episodes** | **18.094** | **0** | **0.14** |
|  | **Ambulatory Counts** | **21.666** | **0** | **0.163** |
|  | Time Spent in Stereotypic Circling | 1.47 | 0.228 | 0.013 |
|  | Incidence of Vertical Stretch Posture | 0.003 | 0.96 | 0 |
|  | *Time Spent in Vertical Stretch Posture* | *2.795* | *0.097* | *0.025* |
|  | Time in Center Zone | 0.084 | 0.772 | 0.001 |
|  | Number of Center Zone Entries | 0.71 | 0.401 | 0.006 |
|  | Number of Clockwise Reversals | 0.255 | 0.614 | 0.002 |
|  | Number of Counter-Clockwise Reversals | 1.746 | 0.189 | 0.015 |
|  | **Ambulatory Distance** | **19.035** | **0** | **0.146** |
|  | **Average Velocity** | **4.76** | **0.031** | **0.041** |
|  | Ambulatory Time | 24.905 | 0 | 0.183 |
|  | **Stereotypic Counts** | **0.064** | **0.8** | **0.001** |
|  | **Resting Time** | **19.284** | **0** | **0.148** |
| Gavage_Treatment | **Jump Counts** | **6.315** | **0.013** | **0.054** |
|  | *Jump Time* | *3.611* | *0.06* | *0.032* |
|  | Ambulatory Episodes | 1.694 | 0.196 | 0.015 |
|  | *Ambulatory Counts* | *3.031* | *0.084* | *0.027* |
|  | **Time Spent in Stereotypic Circling** | **3.911** | **0.05** | **0.034** |
|  | **Incidence of Vertical Stretch Posture** | **4.645** | **0.033** | **0.04** |
|  | Time Spent in Vertical Stretch Posture | 1.982 | 0.162 | 0.018 |
|  | Time in Center Zone | 0.601 | 0.44 | 0.005 |
|  | Number of Center Zone Entries | 5.69 | 0.019 | 0.049 |
|  | Number of Clockwise Reversals | 0.159 | 0.691 | 0.001 |
|  | Number of Counter-Clockwise Reversals | 1.746 | 0.189 | 0.015 |
|  | *Ambulatory Distance* | *2.866* | *0.093* | *0.025* |
|  | Average Velocity | 1.242 | 0.267 | 0.011 |
|  | Ambulatory Time | 1.684 | 0.197 | 0.015 |
|  | **Stereotypic Counts** | **5.114** | **0.026** | **0.044** |
|  | *Resting Time* | *3.507* | *0.064* | *0.031* |
| Naloxone_Treatment | Jump Counts | 1.224 | 0.271 | 0.011 |
|  | Jump Time | 0.051 | 0.821 | 0 |
|  | Ambulatory Episodes | 1.153 | 0.285 | 0.01 |
|  | Ambulatory Counts | 1.457 | 0.23 | 0.013 |
|  | Time Spent in Stereotypic Circling | 0.03 | 0.862 | 0 |
|  | Incidence of Vertical Stretch Posture | 0.565 | 0.454 | 0.005 |
|  | Time Spent in Vertical Stretch Posture | 1.982 | 0.162 | 0.018 |
|  | Time in Center Zone | 0.109 | 0.742 | 0.001 |
|  | Number of Center Zone Entries | 0.214 | 0.645 | 0.002 |
|  | Number of Clockwise Reversals | 0.374 | 0.542 | 0.003 |
|  | Number of Counter-Clockwise Reversals | 0.03 | 0.863 | 0 |
|  | Ambulatory Distance | 1.572 | 0.213 | 0.014 |
|  | Average Velocity | 1.04 | 0.31 | 0.009 |
|  | Ambulatory Time | 1.228 | 0.27 | 0.011 |
|  | Stereotypic Counts | 0.033 | 0.857 | 0 |
|  | Resting Time | 0.673 | 0.414 | 0.006 |
| LPSRS_Treatment | Jump Counts | 0.818 | 0.368 | 0.007 |
|  | Jump Time | 0.152 | 0.698 | 0.001 |
|  | Ambulatory Episodes | 0.272 | 0.603 | 0.002 |
|  | Ambulatory Counts | 0.223 | 0.638 | 0.002 |
|  | Time Spent in Stereotypic Circling | 1.189 | 0.278 | 0.011 |
|  | Incidence of Vertical Stretch Posture | 0.565 | 0.454 | 0.005 |
|  | Time Spent in Vertical Stretch Posture | 0.031 | 0.861 | 0 |
|  | Time in Center Zone | 1.932 | 0.167 | 0.017 |
|  | Number of Center Zone Entries | 0.453 | 0.502 | 0.004 |
|  | Number of Clockwise Reversals | 0.035 | 0.853 | 0 |
|  | Number of Counter-Clockwise Reversals | 0.991 | 0.322 | 0.009 |
|  | Ambulatory Distance | 0.313 | 0.577 | 0.003 |
|  | Average Velocity | 2.918 | 0.09 | 0.026 |
|  | Ambulatory Time | 0.52 | 0.472 | 0.005 |
|  | Stereotypic Counts | 0.463 | 0.498 | 0.004 |
|  | Resting Time | 0.656 | 0.42 | 0.006 |
| Sex * | Jump Counts | 0.221 | 0.639 | 0.002 |
| Gavage_Treatment | Jump Time | 0.051 | 0.822 | 0 |
|  | Ambulatory Episodes | 0.831 | 0.364 | 0.007 |
|  | Ambulatory Counts | 1.029 | 0.313 | 0.009 |
|  | Time Spent in Stereotypic Circling | 0.015 | 0.902 | 0 |
|  | Incidence of Vertical Stretch Posture | 2.113 | 0.149 | 0.019 |
|  | Time Spent in Vertical Stretch Posture | 1.309 | 0.255 | 0.012 |
|  | Time in Center Zone | 1.975 | 0.163 | 0.017 |
|  | Number of Center Zone Entries | 0.414 | 0.521 | 0.004 |
|  | Number of Clockwise Reversals | 0.159 | 0.691 | 0.001 |
|  | Number of Counter-Clockwise Reversals | 0.288 | 0.593 | 0.003 |
|  | Ambulatory Distance | 0.631 | 0.429 | 0.006 |
|  | Average Velocity | 0.056 | 0.814 | 0.001 |
|  | Ambulatory Time | 0.802 | 0.372 | 0.007 |
|  | Stereotypic Counts | 0.015 | 0.904 | 0 |
|  | Resting Time | 0.455 | 0.501 | 0.004 |
| Sex * | **Jump Counts** | **7.133** | **0.009** | **0.06** |
| Naloxone_Treatment | Jump Time | 2.137 | 0.147 | 0.019 |
|  | **Ambulatory Episodes** | **10.018** | **0.002** | **0.083** |
|  | **Ambulatory Counts** | **7.888** | **0.006** | **0.066** |
|  | **Time Spent in Stereotypic Circling** | **6.015** | **0.016** | **0.051** |
|  | Incidence of Vertical Stretch Posture | 2.113 | 0.149 | 0.019 |
|  | Time Spent in Vertical Stretch Posture | 0.379 | 0.539 | 0.003 |
|  | Time in Center Zone | 0.133 | 0.716 | 0.001 |
|  | Number of Center Zone Entries | 0.069 | 0.793 | 0.001 |
|  | Number of Clockwise Reversals | 0.968 | 0.327 | 0.009 |
|  | Number of Counter-Clockwise Reversals | 0.374 | 0.542 | 0.003 |
|  | **Ambulatory Distance** | **7.525** | **0.007** | **0.063** |
|  | **Average Velocity** | **5.315** | **0.023** | **0.046** |
|  | **Ambulatory Time** | **7.326** | **0.008** | **0.062** |
|  | **Stereotypic Counts** | **7.635** | **0.007** | **0.064** |
|  | **Resting Time** | **9.058** | **0.003** | **0.075** |
| Sex * | Jump Counts | 0.981 | 0.324 | 0.009 |
| LPSRS_Treatment | Jump Time | 0.122 | 0.728 | 0.001 |
|  | Ambulatory Episodes | 0.723 | 0.397 | 0.006 |
|  | Ambulatory Counts | 0.596 | 0.442 | 0.005 |
|  | Time Spent in Stereotypic Circling | 0.188 | 0.665 | 0.002 |
|  | Incidence of Vertical Stretch Posture | 2.113 | 0.149 | 0.019 |
|  | Time Spent in Vertical Stretch Posture | 0.07 | 0.792 | 0.001 |
|  | Time in Center Zone | 1.349 | 0.248 | 0.012 |
|  | Number of Center Zone Entries | 0.099 | 0.754 | 0.001 |
|  | Number of Clockwise Reversals | 0.595 | 0.442 | 0.005 |
|  | Number of Counter-Clockwise Reversals | 0.15 | 0.699 | 0.001 |
|  | Ambulatory Distance | 0.578 | 0.449 | 0.005 |
|  | *Average Velocity* | *2.819* | *0.096* | *0.025* |
|  | Ambulatory Time | 0.633 | 0.428 | 0.006 |
|  | Stereotypic Counts | 0.27 | 0.604 | 0.002 |
|  | Resting Time | 0.618 | 0.434 | 0.006 |
| Gavage_Treatment * | Jump Counts | 0.632 | 0.428 | 0.006 |
| Naloxone_Treatment | Jump Time | 0.611 | 0.436 | 0.005 |
|  | Ambulatory Episodes | 0.023 | 0.879 | 0 |
|  | Ambulatory Counts | 0.122 | 0.728 | 0.001 |
|  | Time Spent in Stereotypic Circling | 0.141 | 0.708 | 0.001 |
|  | Incidence of Vertical Stretch Posture | 0.425 | 0.516 | 0.004 |
|  | Time Spent in Vertical Stretch Posture | 0.279 | 0.599 | 0.003 |
|  | Time in Center Zone | 0.03 | 0.862 | 0 |
|  | Number of Center Zone Entries | 0.075 | 0.784 | 0.001 |
|  | Number of Clockwise Reversals | 0.68 | 0.411 | 0.006 |
|  | Number of Counter-Clockwise Reversals | 0.15 | 0.699 | 0.001 |
|  | Ambulatory Distance | 0.124 | 0.726 | 0.001 |
|  | Average Velocity | 2.168 | 0.144 | 0.019 |
|  | Ambulatory Time | 0.098 | 0.761 | 0.001 |
|  | Stereotypic Counts | 0.119 | 0.731 | 0.001 |
|  | Resting Time | 0.218 | 0.642 | 0.002 |
| Gavage_Treatment * | Jump Counts | 0.571 | 0.451 | 0.005 |
| LPSRS_Treatment | Jump Time | 0.376 | 0.541 | 0.003 |
|  | Ambulatory Episodes | 0.44 | 0.509 | 0.004 |
|  | Ambulatory Counts | 0.234 | 0.629 | 0.002 |
|  | Time Spent in Stereotypic Circling | 0.222 | 0.638 | 0.002 |
|  | Incidence of Vertical Stretch Posture | 0.425 | 0.516 | 0.004 |
|  | Time Spent in Vertical Stretch Posture | 0 | 1 | 0 |
|  | Time in Center Zone | 0.68 | 0.411 | 0.006 |
|  | Number of Center Zone Entries | 1.392 | 0.241 | 0.012 |
|  | Number of Clockwise Reversals | 0.68 | 0.411 | 0.006 |
|  | Number of Counter-Clockwise Reversals | 0.918 | 0.34 | 0.008 |
|  | Ambulatory Distance | 0.367 | 0.546 | 0.003 |
|  | Average Velocity | 0.122 | 0.727 | 0.001 |
|  | Ambulatory Time | 0.479 | 0.49 | 0.004 |
|  | Stereotypic Counts | 0.157 | 0.693 | 0.001 |
|  | Resting Time | 0.579 | 0.448 | 0.005 |
| Naloxone_Treatment * | Jump Counts | 0.02 | 0.887 | 0 |
| LPSRS_Treatment | Jump Time | 0.002 | 0.963 | 0 |
|  | Ambulatory Episodes | 1.004 | 0.318 | 0.009 |
|  | Ambulatory Counts | 0.431 | 0.513 | 0.004 |
|  | Time Spent in Stereotypic Circling | 0.164 | 0.687 | 0.001 |
|  | Incidence of Vertical Stretch Posture | 0.425 | 0.516 | 0.004 |
|  | Time Spent in Vertical Stretch Posture | 0.031 | 0.861 | 0 |
|  | Time in Center Zone | 0.174 | 0.677 | 0.002 |
|  | Number of Center Zone Entries | 1.062 | 0.305 | 0.009 |
|  | Number of Clockwise Reversals | 0.018 | 0.894 | 0 |
|  | Number of Counter-Clockwise Reversals | 0.26 | 0.611 | 0.002 |
|  | Ambulatory Distance | 0.305 | 0.582 | 0.003 |
|  | Average Velocity | 0.611 | 0.436 | 0.005 |
|  | Ambulatory Time | 0.642 | 0.425 | 0.006 |
|  | Stereotypic Counts | 0.224 | 0.637 | 0.002 |
|  | Resting Time | 0.538 | 0.465 | 0.005 |
| Sex * | Jump Counts | 1.846 | 0.177 | 0.016 |
| Gavage_Treatment * | Jump Time | 0.328 | 0.568 | 0.003 |
| Naloxone_Treatment | Ambulatory Episodes | 0.733 | 0.394 | 0.007 |
|  | Ambulatory Counts | 1.066 | 0.304 | 0.01 |
|  | Time Spent in Stereotypic Circling | 0.011 | 0.916 | 0 |
|  | Incidence of Vertical Stretch Posture | 0.003 | 0.96 | 0 |
|  | Time Spent in Vertical Stretch Posture | 0.379 | 0.539 | 0.003 |
|  | Time in Center Zone | 0.369 | 0.545 | 0.003 |
|  | Number of Center Zone Entries | 0.013 | 0.908 | 0 |
|  | Number of Clockwise Reversals | 0.018 | 0.894 | 0 |
|  | Number of Counter-Clockwise Reversals | 0.387 | 0.535 | 0.003 |
|  | Ambulatory Distance | 0.675 | 0.413 | 0.006 |
|  | Average Velocity | 0.199 | 0.656 | 0.002 |
|  | Ambulatory Time | 0.611 | 0.436 | 0.005 |
|  | Stereotypic Counts | 0.143 | 0.706 | 0.001 |
|  | Resting Time | 0.421 | 0.518 | 0.004 |
| Sex * | Jump Counts | 0.634 | 0.427 | 0.006 |
| Gavage_Treatment * | Jump Time | 0.771 | 0.382 | 0.007 |
| LPSRS_Treatment | Ambulatory Episodes | 0.531 | 0.468 | 0.005 |
|  | Ambulatory Counts | 0.351 | 0.555 | 0.003 |
|  | Time Spent in Stereotypic Circling | 0.082 | 0.775 | 0.001 |
|  | Incidence of Vertical Stretch Posture | 0.003 | 0.96 | 0 |
|  | Time Spent in Vertical Stretch Posture | 0.194 | 0.661 | 0.002 |
|  | Time in Center Zone | 0.757 | 0.386 | 0.007 |
|  | Number of Center Zone Entries | 0.001 | 0.981 | 0 |
|  | Number of Clockwise Reversals | 0.018 | 0.894 | 0 |
|  | Number of Counter-Clockwise Reversals | 0.329 | 0.567 | 0.003 |
|  | Ambulatory Distance | 0.448 | 0.505 | 0.004 |
|  | Average Velocity | 0.172 | 0.679 | 0.002 |
|  | Ambulatory Time | 0.409 | 0.524 | 0.004 |
|  | Stereotypic Counts | 0.007 | 0.932 | 0 |
|  | Resting Time | 0.287 | 0.593 | 0.003 |
| Sex * | Jump Counts | 0.132 | 0.717 | 0.001 |
| Naloxone_Treatment * | Jump Time | 0.034 | 0.854 | 0 |
| LPSRS_Treatment | Ambulatory Episodes | 0.351 | 0.555 | 0.003 |
|  | Ambulatory Counts | 0.16 | 0.689 | 0.001 |
|  | Time Spent in Stereotypic Circling | 0.611 | 0.436 | 0.005 |
|  | Incidence of Vertical Stretch Posture | 1.831 | 0.179 | 0.016 |
|  | Time Spent in Vertical Stretch Posture | 0.379 | 0.539 | 0.003 |
|  | Time in Center Zone | 0.009 | 0.925 | 0 |
|  | Number of Center Zone Entries | 3.993 | 0.048 | 0.035 |
|  | Number of Clockwise Reversals | 0.595 | 0.442 | 0.005 |
|  | Number of Counter-Clockwise Reversals | 1.204 | 0.275 | 0.011 |
|  | Ambulatory Distance | 0.133 | 0.716 | 0.001 |
|  | Average Velocity | 0.046 | 0.83 | 0 |
|  | Ambulatory Time | 0.175 | 0.677 | 0.002 |
|  | Stereotypic Counts | 0.432 | 0.512 | 0.004 |
|  | Resting Time | 0.336 | 0.564 | 0.003 |
| Gavage_Treatment * | Jump Counts | 1.445 | 0.232 | 0.013 |
| Naloxone_Treatment * | Jump Time | 1 | 0.32 | 0.009 |
| LPSRS_Treatment | Ambulatory Episodes | 1.24 | 0.268 | 0.011 |
|  | Ambulatory Counts | 0.376 | 0.541 | 0.003 |
|  | Time Spent in Stereotypic Circling | 2.32 | 0.131 | 0.02 |
|  | Incidence of Vertical Stretch Posture | 0.565 | 0.454 | 0.005 |
|  | Time Spent in Vertical Stretch Posture | 1.115 | 0.293 | 0.01 |
|  | Time in Center Zone | 0.117 | 0.733 | 0.001 |
|  | Number of Center Zone Entries | 0.133 | 0.716 | 0.001 |
|  | Number of Clockwise Reversals | 0.204 | 0.652 | 0.002 |
|  | Number of Counter-Clockwise Reversals | 0.104 | 0.747 | 0.001 |
|  | Ambulatory Distance | 0.697 | 0.406 | 0.006 |
|  | Average Velocity | 2.653 | 0.106 | 0.023 |
|  | Ambulatory Time | 0.503 | 0.48 | 0.005 |
|  | Stereotypic Counts | 2.499 | 0.117 | 0.022 |
|  | Resting Time | 1.323 | 0.253 | 0.012 |
| Sex * | Jump Counts | 0 | 0.994 | 0 |
| Gavage_Treatment * | Jump Time | 0.159 | 0.691 | 0.001 |
| Naloxone_Treatment * | Ambulatory Episodes | 0.859 | 0.356 | 0.008 |
| LPSRS_Treatment | Ambulatory Counts | 0.51 | 0.477 | 0.005 |
|  | Time Spent in Stereotypic Circling | 1.305 | 0.256 | 0.012 |
|  | Incidence of Vertical Stretch Posture | 0.003 | 0.96 | 0 |
|  | *Time Spent in Vertical Stretch Posture* | *3.415* | *0.067* | *0.03* |
|  | Time in Center Zone | 0.009 | 0.926 | 0 |
|  | Number of Center Zone Entries | 0.759 | 0.386 | 0.007 |
|  | Number of Clockwise Reversals | 1.189 | 0.278 | 0.011 |
|  | Number of Counter-Clockwise Reversals | 0.13 | 0.719 | 0.001 |
|  | Ambulatory Distance | 0.654 | 0.42 | 0.006 |
|  | Average Velocity | 0.01 | 0.921 | 0 |
|  | Ambulatory Time | 0.889 | 0.348 | 0.008 |
|  | Stereotypic Counts | 1.368 | 0.245 | 0.012 |
|  | Resting Time | 1.248 | 0.266 | 0.011 |

Legend: Individual ANOVAs on outcome variables measured in Experiment 2. Significant results are boldfaced. Trends are italicized. F values are indicated in the “F” column, p values are indicated in the "Sig." column and effect sizes (partial eta squared) are indicated in the "Partial η^2" column. For each ANOVA, hypothesis degrees of freedom is 1 and error degrees of freedom is 111.
