# Supplementary material for: Effects of gut-derived endotoxin on anxiety-like and repetitive behaviors in male and female mice
Source: Biol Sex Differ. 2018 Jan 19;9:7. doi: 10.1186/s13293-018-0166-x (PMC5775597; doi:10.1186/s13293-018-0166-x)
Supplement: Supplementary file 4 — Title: Original classification and cross-validation of discriminant functions for Experiment 2 for cases grouped by gavage treatment and sex. Legend: Validation of discriminant functions for Experiment 2, for cases grouped by gavage treatment and sex, by original case classification and leave-one-out cross validation. 64.6% of the original grouped cases are correctly classified by the discriminant functions. In the leave-one-out cross-validation test, the discriminant functions are recalculated excluding one case, and all cases are recalculated. This algorithm is repeated for the exclusion of each case. In the leave-one-out test, 45.7% of cross-validated grouped cases were correctly classified. (DOCX 14 kb) [file 13293_2018_166_MOESM4_ESM.docx]

Additional file 4 Table S4: Title: Original classification and cross-validation of discriminant functions for Experiment 2 for cases grouped by gavage treatment and sex.

| **Classification Results^a,c^** | | | | | | | |
| --- | --- | --- | --- | --- | --- | --- | --- |
|  |  | SexByGavage | Predicted Group Membership | | | | Total |
|  |  |  | Male Saline | Male LPS | Female Saline | Female LPS |  |
| Original | Count | Male Saline | 22 | 9 | 1 | 0 | 32 |
|  |  | Male LPS | 9 | 20 | 1 | 2 | 32 |
|  |  | Female Saline | 3 | 2 | 21 | 5 | 31 |
|  |  | Female LPS | 0 | 4 | 9 | 19 | 32 |
|  | % | Male Saline | 68.8 | 28.1 | 3.1 | .0 | 100.0 |
|  |  | Male LPS | 28.1 | 62.5 | 3.1 | 6.3 | 100.0 |
|  |  | Female Saline | 9.7 | 6.5 | 67.7 | 16.1 | 100.0 |
|  |  | Female LPS | .0 | 12.5 | 28.1 | 59.4 | 100.0 |
| Cross-validated^b^ | Count | Male Saline | 15 | 13 | 3 | 1 | 32 |
|  |  | Male LPS | 13 | 14 | 2 | 3 | 32 |
|  |  | Female Saline | 5 | 3 | 14 | 9 | 31 |
|  |  | Female LPS | 1 | 5 | 11 | 15 | 32 |
|  | % | Male Saline | 46.9 | 40.6 | 9.4 | 3.1 | 100.0 |
|  |  | Male LPS | 40.6 | 43.8 | 6.3 | 9.4 | 100.0 |
|  |  | Female Saline | 16.1 | 9.7 | 45.2 | 29.0 | 100.0 |
|  |  | Female LPS | 3.1 | 15.6 | 34.4 | 46.9 | 100.0 |

Legend: Validation of discriminant functions for Experiment 2, for cases grouped by gavage treatment and sex, by original case classification and leave-one-out cross validation. 64.6% of the original grouped cases are correctly classified by the discriminant functions. In the leave-one-out cross-validation test, the discriminant functions are recalculated excluding one case, and all cases are recalculated. This algorithm is repeated for the exclusion of each case. In the leave-one-out test, 45.7% of cross-validated grouped cases were correctly classified.
