# Supplementary material for: Effects of gut-derived endotoxin on anxiety-like and repetitive behaviors in male and female mice
Source: Biol Sex Differ. 2018 Jan 19;9:7. doi: 10.1186/s13293-018-0166-x (PMC5775597; doi:10.1186/s13293-018-0166-x)
Supplement: Supplementary file 5 — Title: Original classification and cross-validation of discriminant functions for Experiment 1. Legend: Validation of discriminant functions for Experiment 2, for cases grouped by (+)-naloxone treatment and sex, by original case classification and leave-one-out cross validation. 65.4% of the original grouped cases are correctly classified by the discriminant functions. In the leave-one-out cross-validation test, the discriminant functions are recalculated excluding one case, and all cases are recalculated. This algorithm is repeated for the exclusion of each case. In the leave-one-out test, 48.0% of cross-validated grouped cases were correctly classified. (DOCX 14 kb) [file 13293_2018_166_MOESM5_ESM.docx]

Additional file 5: Table S5: Title: Original classification and cross-validation of discriminant functions for Experiment 1.

| **Classification Results^a,c^** | | | | | | | |
| --- | --- | --- | --- | --- | --- | --- | --- |
|  |  | SexByNaloxone | Predicted Group Membership | | | | Total |
|  |  |  | Male Saline | Male (+)-naloxone | Female Saline | Female (+)-naloxone |  |
| Original | Count | Male Saline | 20 | 10 | 2 | 0 | 32 |
|  |  | Male (+)-naloxone | 9 | 22 | 1 | 0 | 32 |
|  |  | Female Saline | 2 | 3 | 20 | 7 | 32 |
|  |  | Female (+)-naloxone | 2 | 1 | 7 | 21 | 31 |
|  | % | Male Saline | 62.5 | 31.3 | 6.3 | .0 | 100.0 |
|  |  | Male (+)-naloxone | 28.1 | 68.8 | 3.1 | .0 | 100.0 |
|  |  | Female Saline | 6.3 | 9.4 | 62.5 | 21.9 | 100.0 |
|  |  | Female (+)-naloxone | 6.5 | 3.2 | 22.6 | 67.7 | 100.0 |
| Cross-validated^b^ | Count | Male Saline | 18 | 11 | 3 | 0 | 32 |
|  |  | Male (+)-naloxone | 15 | 14 | 3 | 0 | 32 |
|  |  | Female Saline | 5 | 3 | 14 | 10 | 32 |
|  |  | Female (+)-naloxone | 3 | 2 | 11 | 15 | 31 |
|  | % | Male Saline | 56.3 | 34.4 | 9.4 | .0 | 100.0 |
|  |  | Male (+)-naloxone | 46.9 | 43.8 | 9.4 | .0 | 100.0 |
|  |  | Female Saline | 15.6 | 9.4 | 43.8 | 31.3 | 100.0 |
|  |  | Female (+)-naloxone | 9.7 | 6.5 | 35.5 | 48.4 | 100.0 |

Legend: Validation of discriminant functions for Experiment 2, for cases grouped by (+)-naloxone treatment and sex, by original case classification and leave-one-out cross validation. 65.4% of the original grouped cases are correctly classified by the discriminant functions. In the leave-one-out cross-validation test, the discriminant functions are recalculated excluding one case, and all cases are recalculated. This algorithm is repeated for the exclusion of each case. In the leave-one-out test, 48.0% of cross-validated grouped cases were correctly classified.
