# Supplementary figures and images for: Effects of gut-derived endotoxin on anxiety-like and repetitive behaviors in male and female mice
Source: Biol Sex Differ. 2018 Jan 19;9:7. doi: 10.1186/s13293-018-0166-x (PMC5775597; doi:10.1186/s13293-018-0166-x)

## Slide 1
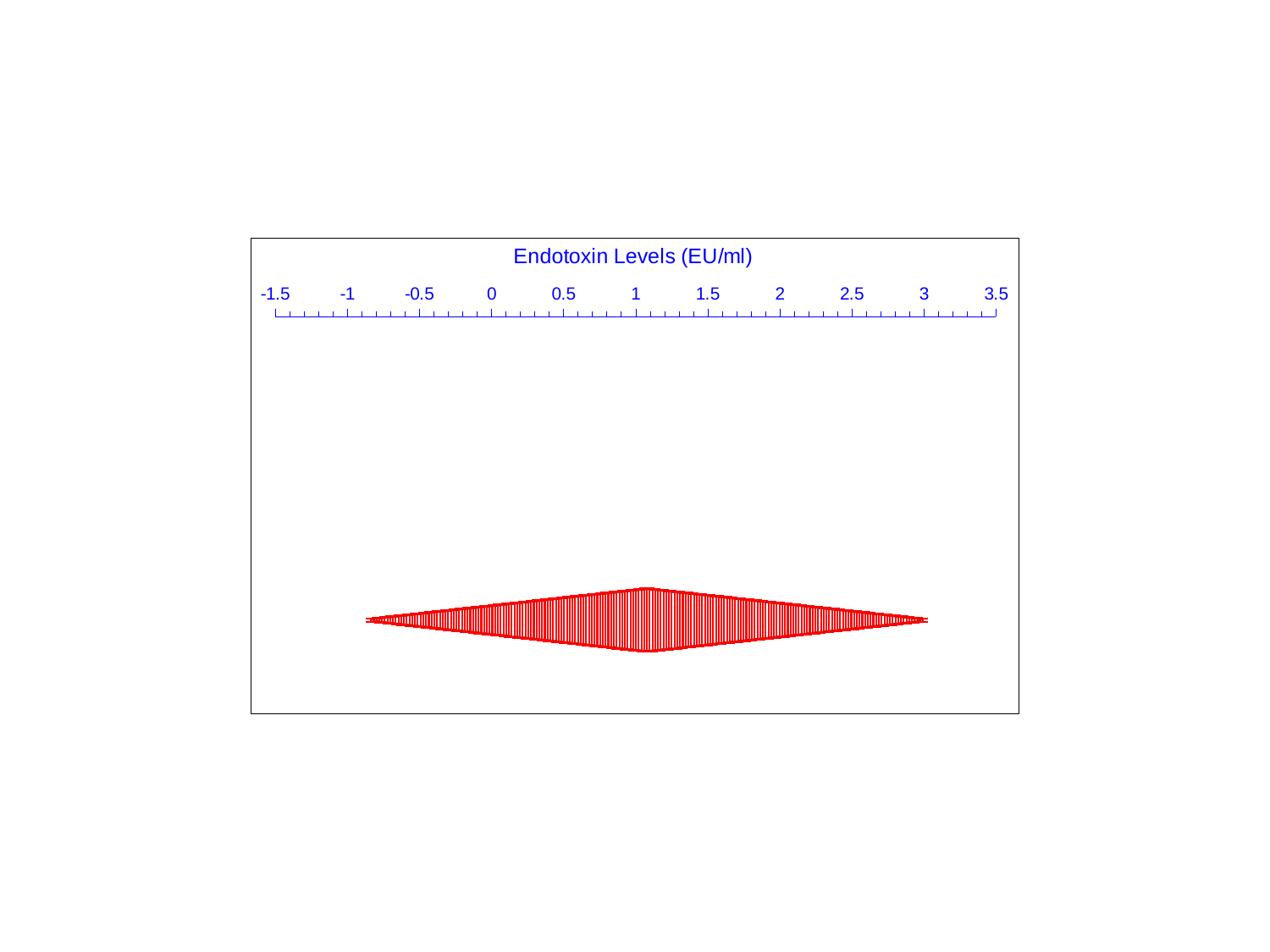

### Chart
| Category | | | | | | | | | | | | | | |
|---|---|---|---|---|---|---|---|---|---|---|---|---|---|---|

Supplement: Supplementary file 6 — Title: Meta-analysis of serum endotoxin levels in WT males of Experiments 1 and 2. Legend: Forest plot of difference in serum endotoxin levels between male WT subjects gavaged with saline or LPS, measured from Experiment 1 (top green bar) and Experiment 2 (bottom green bar). The result of the meta-analysis is indicated by the red diamond. The width of the green bars and the red diamond indicate the range of the 95% confidence intervals for each, with the center representing the mean. WT males not treated with TLR4 antagonists were used for these analyses (total n = 15/group). (PPTX 45 kb) [file 13293_2018_166_MOESM6_ESM.pptx]

## Slide 1
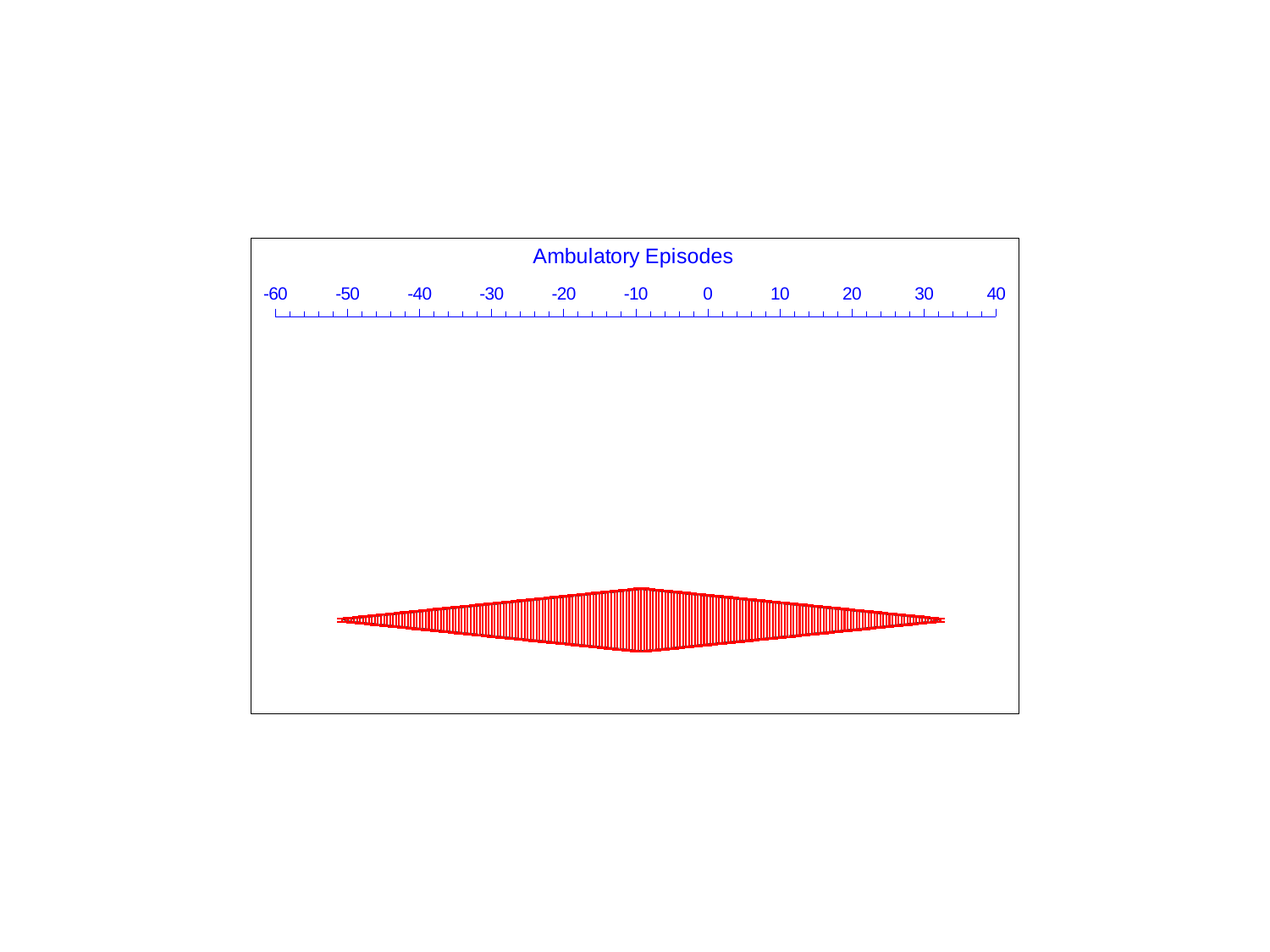

### Chart
| Category | | | | | | | | | | | | | | |
|---|---|---|---|---|---|---|---|---|---|---|---|---|---|---|

Supplement: Supplementary file 7 — Title: Meta-analysis of ambulatory episodes in WT males of Experiments 1 and 2. Legend: Forest plot of difference in ambulatory episodes between male WT subjects gavaged with saline or LPS, measured from Experiment 1 (top green bar) and Experiment 2 (bottom green bar). The result of the meta-analysis is indicated by the red diamond. The width of the green bars and the red diamond indicate the range of the 95% confidence intervals for each, with the center representing the mean. WT males not treated with TLR4 antagonists were used for these analyses (total n = 15/group). See Additional file 18: Table S7 for statistics. (PPTX 45 kb) [file 13293_2018_166_MOESM7_ESM.pptx]

## Slide 1
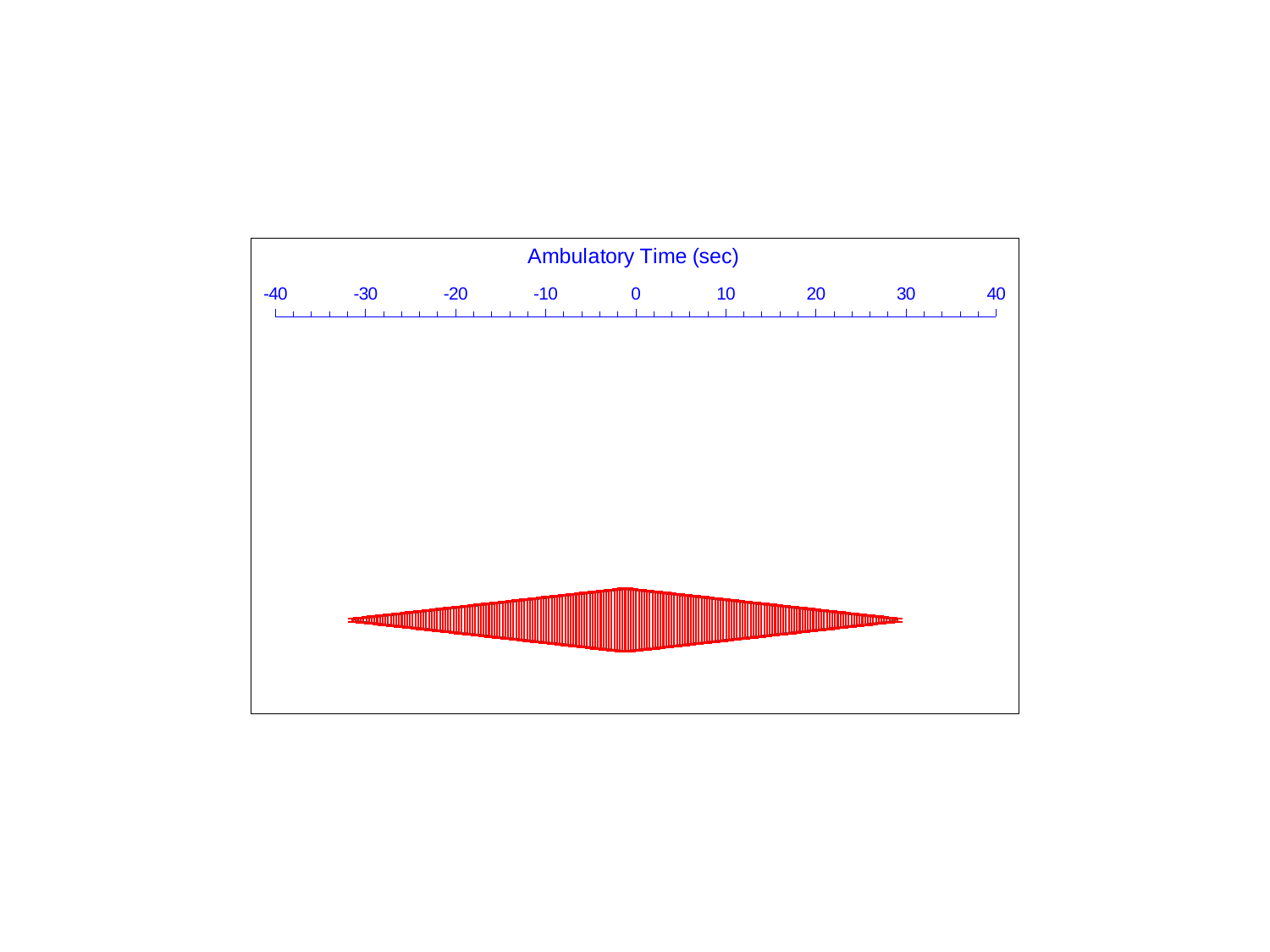

### Chart
| Category | | | | | | | | | | | | | | |
|---|---|---|---|---|---|---|---|---|---|---|---|---|---|---|

Supplement: Supplementary file 8 — Title: Meta-analysis of ambulatory time in WT males of Experiments 1 and 2. Legend: Forest plot of difference in ambulatory time between male WT subjects gavaged with saline or LPS, measured from Experiment 1 (top green bar) and Experiment 2 (bottom green bar). The result of the meta-analysis is indicated by the red diamond. The width of the green bars and the red diamond indicate the range of the 95% confidence intervals for each, with the center representing the mean. WT males not treated with TLR4 antagonists were used for these analyses (total n = 15/group). See Additional file 18: Table S7 for statistics. (PPTX 45 kb) [file 13293_2018_166_MOESM8_ESM.pptx]

## Slide 1
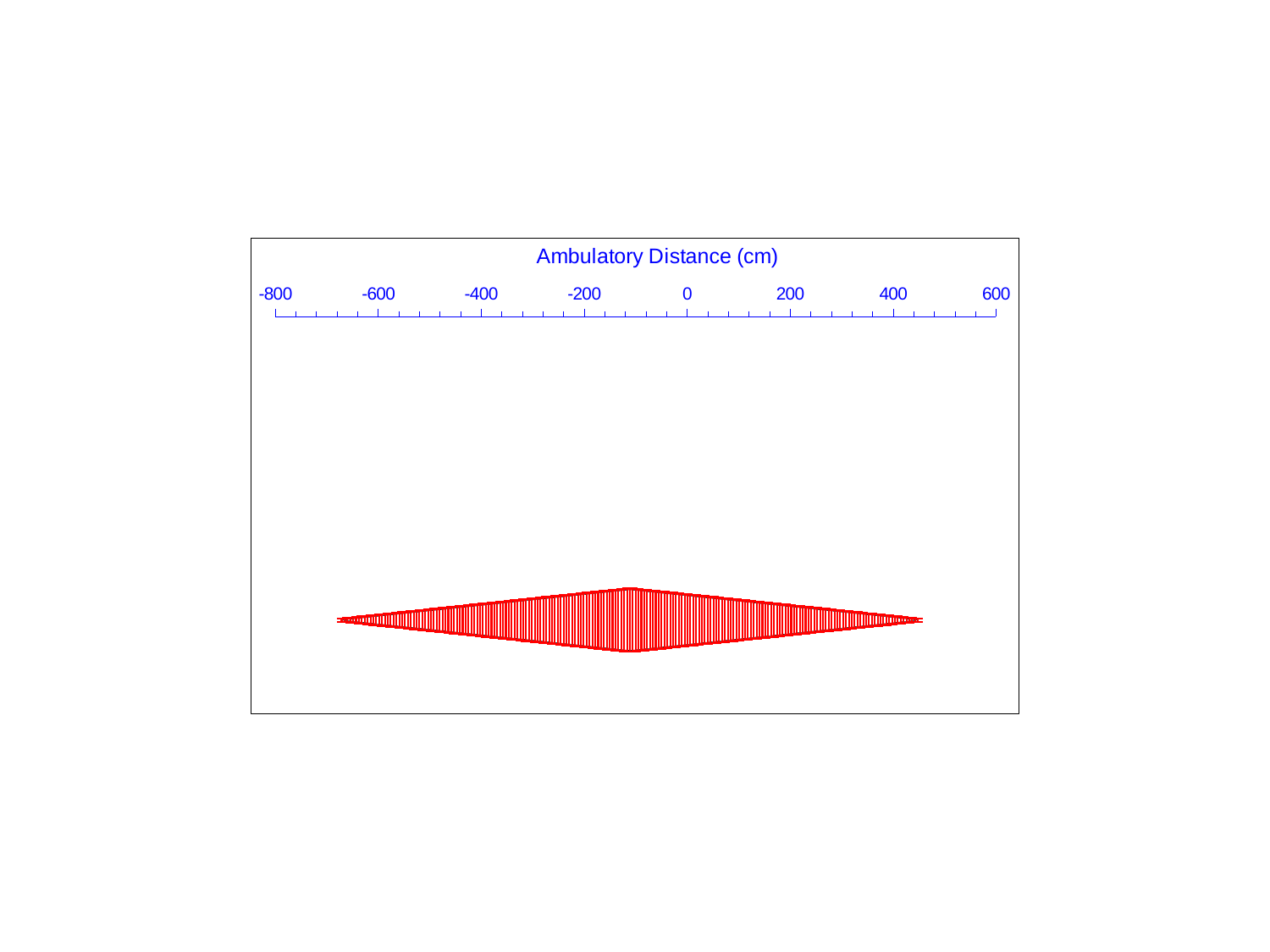

### Chart
| Category | | | | | | | | | | | | | | |
|---|---|---|---|---|---|---|---|---|---|---|---|---|---|---|

Supplement: Supplementary file 9 — Meta-analysis of ambulatory distance in WT males of Experiments 1 and 2. Legend: Forest plot of difference in ambulatory distance between male WT subjects gavaged with saline or LPS, measured from Experiment 1 (top green bar) and Experiment 2 (bottom green bar). The result of the meta-analysis is indicated by the red diamond. The width of the green bars and the red diamond indicate the range of the 95% confidence intervals for each, with the center representing the mean. WT males not treated with TLR4 antagonists were used for these analyses (total n = 15/group). See Additional file 18: Table S7 for statistics. (PPTX 45 kb) [file 13293_2018_166_MOESM9_ESM.pptx]

## Slide 1
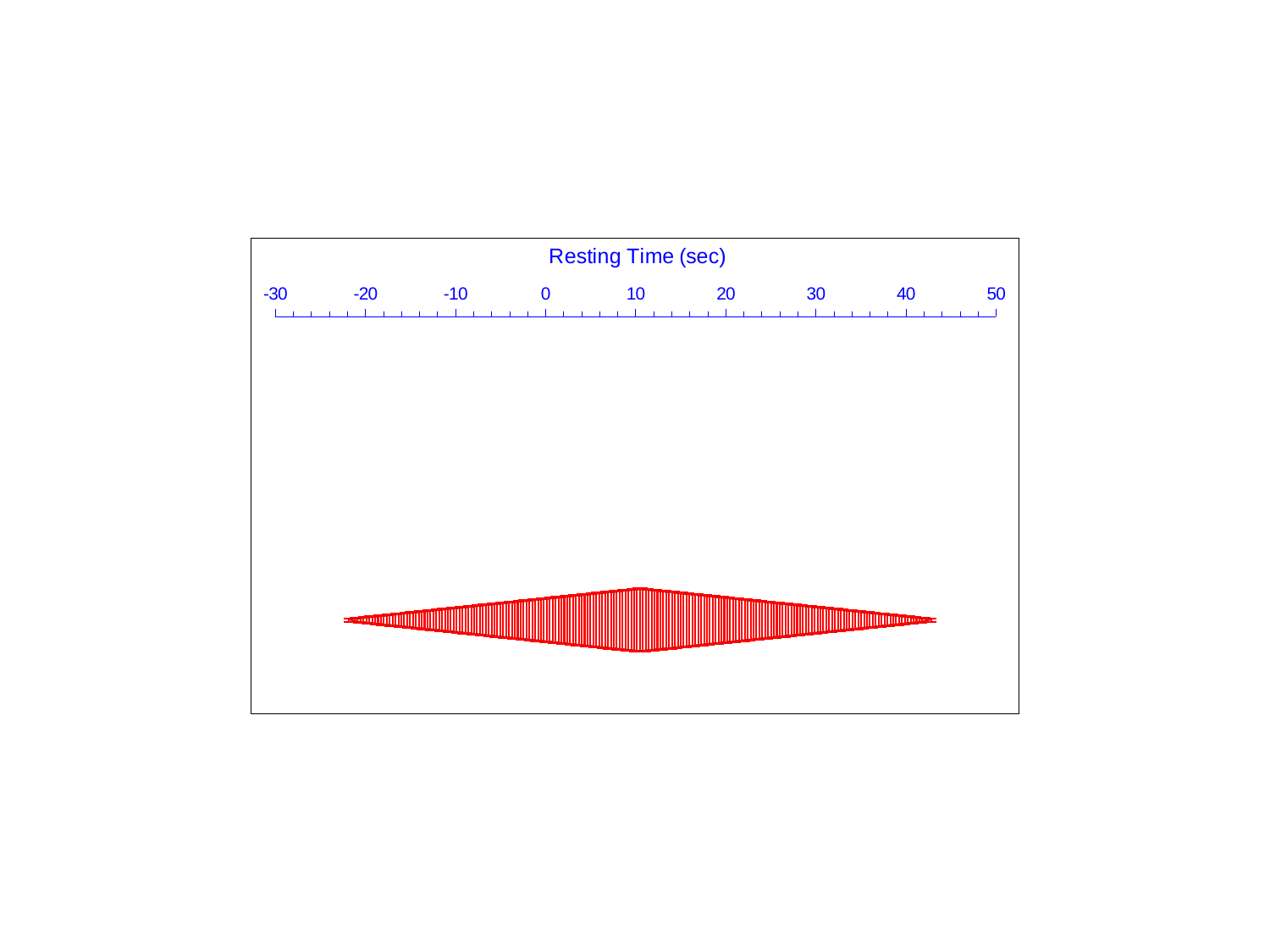

### Chart
| Category | | | | | | | | | | | | | | |
|---|---|---|---|---|---|---|---|---|---|---|---|---|---|---|

Supplement: Supplementary file 10 — Meta-analysis of resting time in WT males of Experiments 1 and 2. Legend: Forest plot of difference in resting time between male WT subjects gavaged with saline or LPS, measured from Experiment 1 (top green bar) and Experiment 2 (bottom green bar). The result of the meta-analysis is indicated by the red diamond. The width of the green bars and the red diamond indicate the range of the 95% confidence intervals for each, with the center representing the mean. WT males not treated with TLR4 antagonists were used for these analyses (total n = 15/group). See Additional file 18: Table S7 for statistics. (PPTX 45 kb) [file 13293_2018_166_MOESM10_ESM.pptx]

## Slide 1
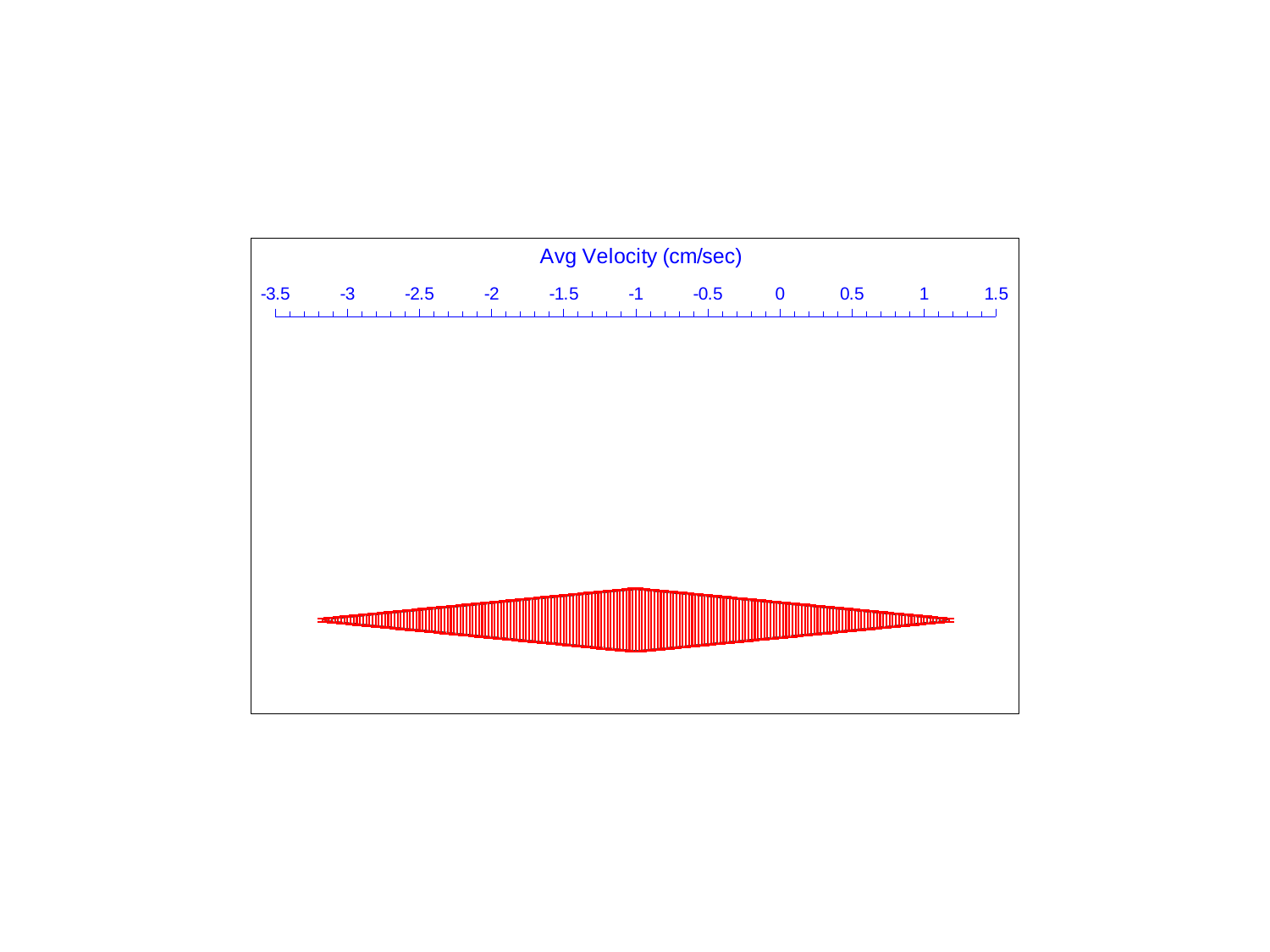

### Chart
| Category | | | | | | | | | | | | | | |
|---|---|---|---|---|---|---|---|---|---|---|---|---|---|---|

Supplement: Supplementary file 11 — Meta-analysis of average velocity in WT males of Experiments 1 and 2. Legend: Forest plot of difference in average velocity between male WT subjects gavaged with saline or LPS, measured from Experiment 1 (top green bar) and Experiment 2 (bottom green bar). The result of the meta-analysis is indicated by the red diamond. The width of the green bars and the red diamond indicate the range of the 95% confidence intervals for each, with the center representing the mean. WT males not treated with TLR4 antagonists were used for these analyses (total n = 15/group). See Additional file 18: Table S7 for statistics. (PPTX 45 kb) [file 13293_2018_166_MOESM11_ESM.pptx]

## Slide 1
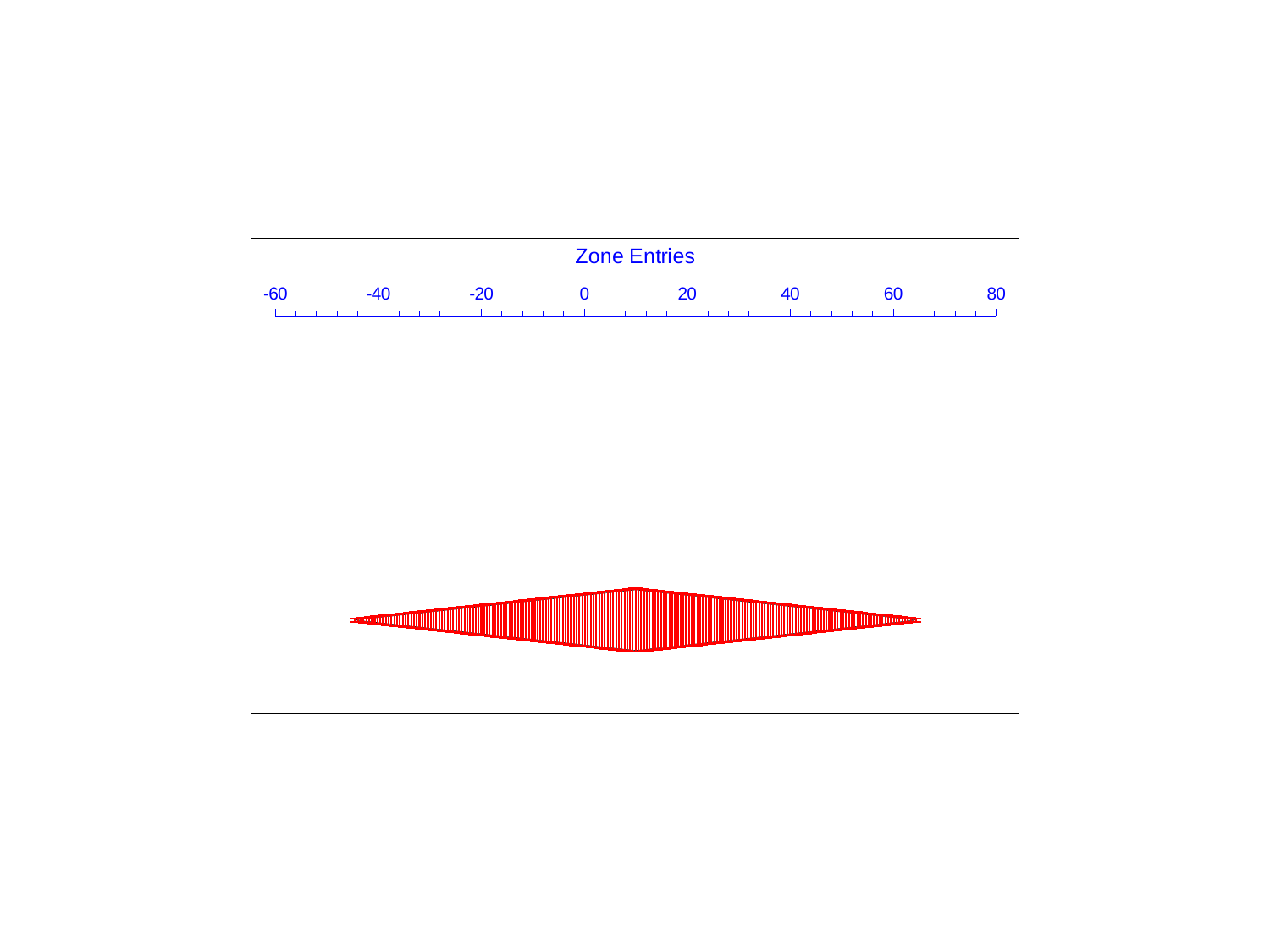

### Chart
| Category | | | | | | | | | | | | | | |
|---|---|---|---|---|---|---|---|---|---|---|---|---|---|---|

Supplement: Supplementary file 12 — Meta-analysis of zone entries in WT males of Experiments 1 and 2. Legend: Forest plot of difference in zone entries between male WT subjects gavaged with saline or LPS, measured from Experiment 1 (top green bar) and Experiment 2 (bottom green bar). The result of the meta-analysis is indicated by the red diamond. The width of the green bars and the red diamond indicate the range of the 95% confidence intervals for each, with the center representing the mean. WT males not treated with TLR4 antagonists were used for these analyses (total n = 15/group). See Additional file 18: Table S7 for statistics. (PPTX 45 kb) [file 13293_2018_166_MOESM12_ESM.pptx]

## Slide 1
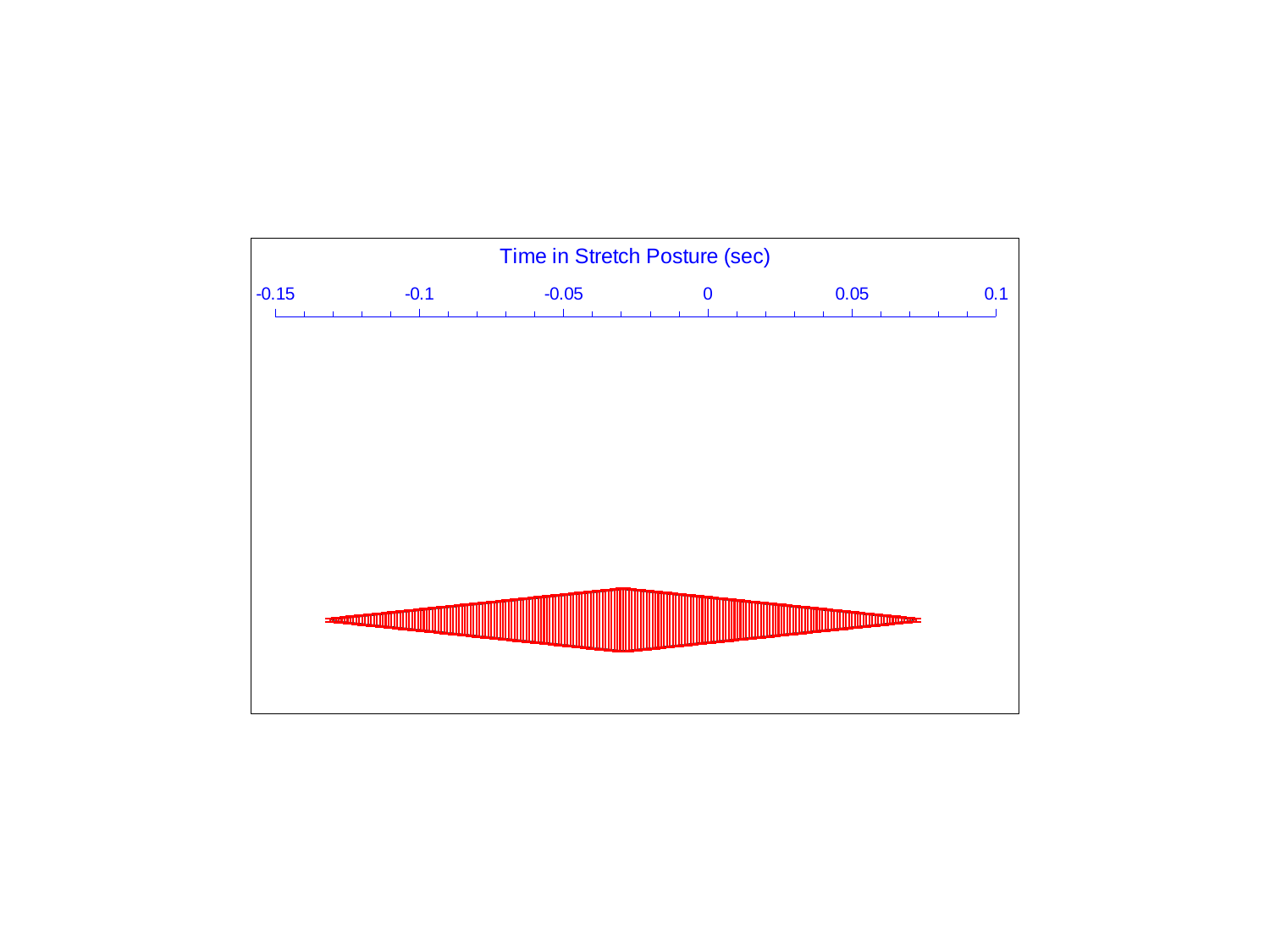

### Chart
| Category | | | | | | | | | | | | | | |
|---|---|---|---|---|---|---|---|---|---|---|---|---|---|---|

Supplement: Supplementary file 13 — Meta-analysis of stretch posture in WT males of Experiments 1 and 2. Legend: Forest plot of difference in time in stretch posture between male WT subjects gavaged with saline or LPS, measured from Experiment 1 (top green bar) and Experiment 2 (bottom green bar). The result of the meta-analysis is indicated by the red diamond. The width of the green bars and the red diamond indicate the range of the 95% confidence intervals for each, with the center representing the mean. WT males not treated with TLR4 antagonists were used for these analyses (total n = 15/group). See Additional file 18: Table S7 for statistics. (PPTX 45 kb) [file 13293_2018_166_MOESM13_ESM.pptx]

## Slide 1
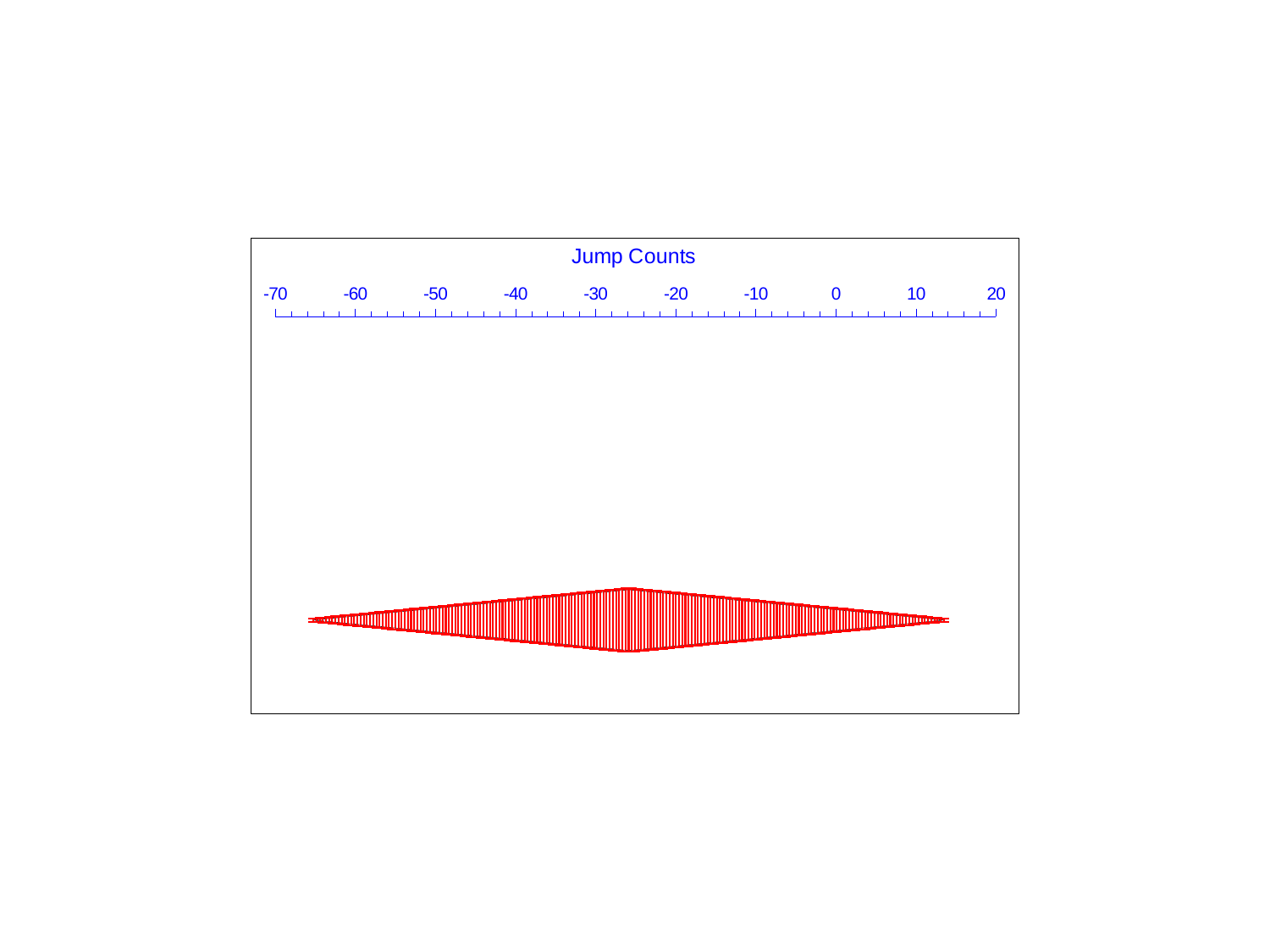

### Chart
| Category | | | | | | | | | | | | | | |
|---|---|---|---|---|---|---|---|---|---|---|---|---|---|---|

Supplement: Supplementary file 14 — Meta-analysis of jump counts in WT males of Experiments 1 and 2. Legend: Forest plot of difference in jump counts between male WT subjects gavaged with saline or LPS, measured from Experiment 1 (top green bar) and Experiment 2 (bottom green bar). The result of the meta-analysis is indicated by the red diamond. The width of the green bars and the red diamond indicate the range of the 95% confidence intervals for each, with the center representing the mean. WT males not treated with TLR4 antagonists were used for these analyses (total n = 15/group). See Additional file 18: Table S7 for statistics. (PPTX 45 kb) [file 13293_2018_166_MOESM14_ESM.pptx]

## Slide 1
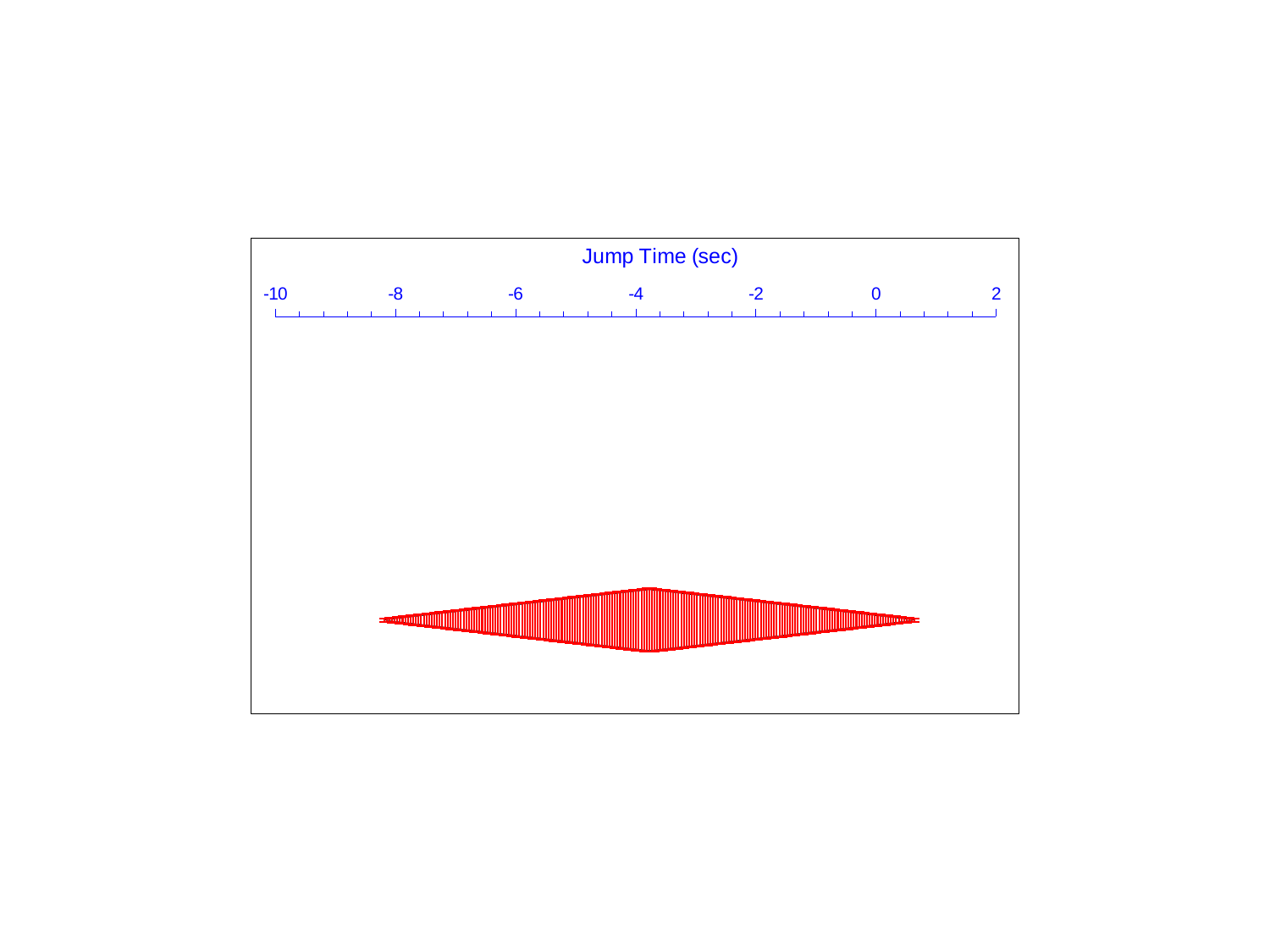

### Chart
| Category | | | | | | | | | | | | | | |
|---|---|---|---|---|---|---|---|---|---|---|---|---|---|---|

Supplement: Supplementary file 15 — Meta-analysis of jump time in WT males of Experiments 1 and 2. Legend: Forest plot of difference in jump time between male WT subjects gavaged with saline or LPS, measured from Experiment 1 (top green bar) and Experiment 2 (bottom green bar). The result of the meta-analysis is indicated by the red diamond. The width of the green bars and the red diamond indicate the range of the 95% confidence intervals for each, with the center representing the mean. WT males not treated with TLR4 antagonists were used for these analyses (total n = 15/group). See Additional file 18: Table S7 for statistics (PPTX 45 kb) [file 13293_2018_166_MOESM15_ESM.pptx]

## Slide 1
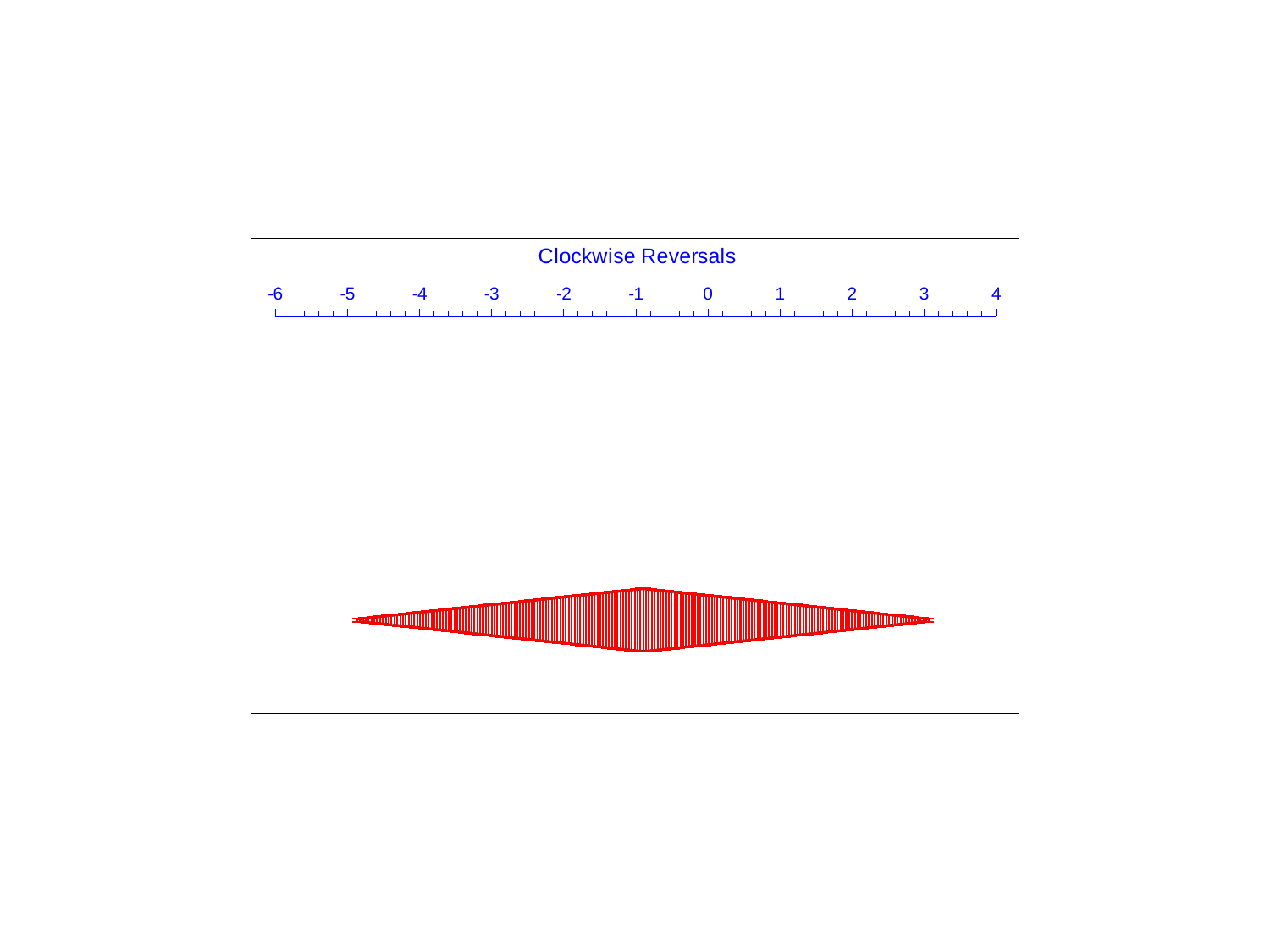

### Chart
| Category | | | | | | | | | | | | | | |
|---|---|---|---|---|---|---|---|---|---|---|---|---|---|---|

Supplement: Supplementary file 16 — Meta-analysis of clockwise reversals in WT males of Experiments 1 and 2. Legend: Forest plot of difference in clockwise reversals between male WT subjects gavaged with saline or LPS, measured from Experiment 1 (top green bar) and Experiment 2 (bottom green bar). The result of the meta-analysis is indicated by the red diamond. The width of the green bars and the red diamond indicate the range of the 95% confidence intervals for each, with the center representing the mean. WT males not treated with TLR4 antagonists were used for these analyses (total n = 15/group). See Additional file 18: Table S7 for statistics. (PPTX 45 kb) [file 13293_2018_166_MOESM16_ESM.pptx]

## Slide 1
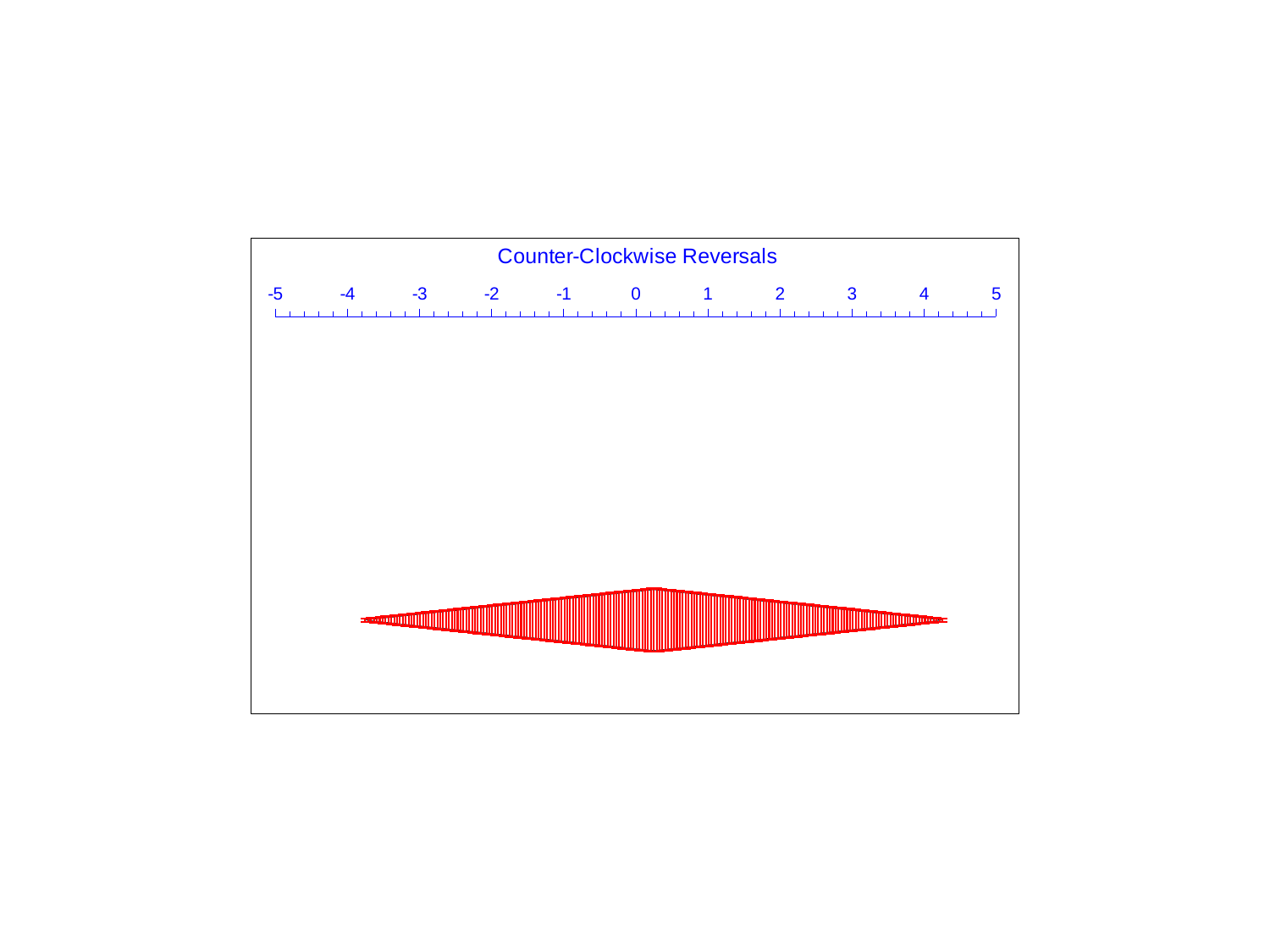

### Chart
| Category | | | | | | | | | | | | | | |
|---|---|---|---|---|---|---|---|---|---|---|---|---|---|---|

Supplement: Supplementary file 17 — Meta-analysis of counter-clockwise reversals in WT males of Experiments 1 and 2. Legend: Forest plot of difference in counter-clockwise reversals between male WT subjects gavaged with saline or LPS, measured from Experiment 1 (top green bar) and Experiment 2 (bottom green bar). The result of the meta-analysis is indicated by the red diamond. The width of the green bars and the red diamond indicate the range of the 95% confidence intervals for each, with the center representing the mean. WT males not treated with TLR4 antagonists were used for these analyses (total n = 15/group). See Additional file 18: Table S7 for statistics. (PPTX 45 kb) [file 13293_2018_166_MOESM17_ESM.pptx]
