# Supplementary material for: Effects of gut-derived endotoxin on anxiety-like and repetitive behaviors in male and female mice
Source: Biol Sex Differ. 2018 Jan 19;9:7. doi: 10.1186/s13293-018-0166-x (PMC5775597; doi:10.1186/s13293-018-0166-x)
Supplement: Supplementary file 18 — Meta-analysis of behavioral outcomes for WT males not treated with TLR4 antagonists (n = 15/group). Legend: 95% confidence interval values for Experiment 1, Experiment 2, and the meta-analysis. UL = Upper Limit. LL = Lower Limit. (DOCX 17 kb) [file 13293_2018_166_MOESM18_ESM.docx]

Additional file 18: Table S7: Meta analysis of behavioral outcomes for WT males not treated with TLR4 antagonists (n=15/group).

| Measure | Mean - Exp 1 | UL - Exp 1 | LL - Exp 1 |
| --- | --- | --- | --- |
| Time in Center Zone (sec) | -35.279 | -49.205 | -8.662 |
| Time Spent in Stereotypic Circling (sec) | -7.507 | -14.94 | -0.075 |
| Stereotypic Counts | -122.29 | -248.4 | 3.827 |
| Ambulatory Counts | 181.43 | -366.53 | 729.388 |
| Ambulatory Episodes | 5.572 | -29.281 | 40.424 |
| Ambulatory Time (sec) | 16.914 | -18.596 | 52.425 |
| Ambulatory Distance (cm) | 235.66 | -489.47 | 960.787 |
| Resting Time (sec) | -6.764 | -42.879 | 29.351 |
| Avg Velocity (cm/sec) | -1.462 | -4.527 | 1.603 |
| Zone Entries | 38.121 | 19.832 | 56.411 |
| Time in Stretch Posture (sec) | -0.093 | -0.391 | 0.206 |
| Jump Counts | -11.143 | -39.197 | 16.911 |
| Jump Time (sec) | -2.65 | -8.241 | 2.941 |
| Clockwise Reversals | -0.429 | -5.304 | 4.447 |
| Counter-Clockwise Reversals | -0.143 | -4.901 | 4.615 |
|  | Mean - Exp 2 | UL - Exp 2 | LL - Exp 2 |
| Time in Center Zone (sec) | -12.075 | -50.105 | 25.955 |
| Time Spent in Stereotypic Circling (sec) | -8.112 | -18.754 | 2.529 |
| Stereotypic Counts | -173 | -355.79 | 9.787 |
| Ambulatory Counts | -219.13 | -536.6 | 98.354 |
| Ambulatory Episodes | -39.5 | -105.7 | 26.705 |
| Ambulatory Time (sec) | -14.338 | -37.113 | 8.438 |
| Ambulatory Distance (cm) | -346.34 | -804.57 | 111.894 |
| Resting Time (sec) | 26.231 | -5.972 | 58.434 |
| Avg Velocity (cm/sec) | 0.24 | -4.106 | 3.626 |
| Zone Entries | -17.5 | -30.366 | -4.635 |
| Time in Stretch Posture (sec) | -0.019 | -0.139 | 0.102 |
| Jump Counts | -53.25 | -109.91 | 3.407 |
| Jump Time (sec) | -7.564 | -17.68 | 2.555 |
| Clockwise Reversals | -3 | -13.19 | 7.19 |
| Counter-Clockwise Reversals | 3 | -9.357 | 15.357 |
|  | Mean - Meta Analysis | UL - Meta Analysis | LL - Meta Analysis |
| Time in Center Zone (sec) | -7.507 | -14.94 | -0.075 |
| Time Spent in Stereotypic Circling (sec) | -7.701 | -13.211 | -2.192 |
| Stereotypic Counts | -138.29 | -232.14 | -44.447 |
| Ambulatory Counts | -70.45 | -449.73 | 308.832 |
| Ambulatory Episodes | -9.233 | -50.721 | 32.256 |
| Ambulatory Time (sec) | -1.157 | -31.406 | 29.091 |
| Ambulatory Distance (cm) | -111.28 | -670.99 | 448.426 |
| Resting Time (sec) | 10.481 | -21.821 | 42.782 |
| Avg Velocity (cm/sec) | -0.999 | -3.173 | 1.174 |
| Zone Entries | 10.001 | -44.504 | 64.505 |
| Time in Stretch Posture (sec) | -0.029 | -0.131 | 0.072 |
| Jump Counts | -25.879 | -65.241 | 13.484 |
| Jump Time (sec) | -3.772 | -8.19 | 0.646 |
| Clockwise Reversals | -0.895 | -4.863 | 3.072 |
| Counter-Clockwise Reversals | 0.252 | -3.75 | 4.254 |

Legend: 95% confidence interval values for Experiment 1, Experiment 2, and the meta analysis. UL=Upper Limit. LL=Lower Limit.
